# Flow cytometric quantification, sorting and sequencing of methanogenic archaea based on F_420_ autofluorescence

**DOI:** 10.1186/s12934-017-0793-7

**Published:** 2017-10-30

**Authors:** Johannes Lambrecht, Nicolas Cichocki, Thomas Hübschmann, Christin Koch, Hauke Harms, Susann Müller

**Affiliations:** Department of Environmental Microbiology, Helmholtz Centre for Environmental Research-UFZ, Permoserstr. 15, 04318 Leipzig, Germany

**Keywords:** Single cell analytics, F_420_, Autofluorescence, Methanogenic archaea, Anaerobic digestion, Biogas, 16S rDNA sequencing, Process monitoring

## Abstract

**Background:**

The widely established production of CH_4_ from renewable biomass in industrial scale anaerobic reactors may play a major role in the future energy supply. It relies on methanogenic archaea as key organisms which represent the bottleneck in the process. The quantitative analysis of these organisms can help to maximize process performance, uncover disturbances before failure, and may ultimately lead to community-based process control schemes. Existing qPCR and fluorescence microscopy-based methods are very attractive but can be cost-intensive and laborious.

**Results:**

In this study we present an autofluorescence-based, flow cytometric method for the fast low-cost quantification of methanogenic archaea in complex microbial communities and crude substrates. The method was applied to a methanogenic enrichment culture (MEC) and digester samples (DS). The methanogenic archaea were quantified using the distinct fluorescence of their cofactor F_420_ in a range from 3.7 × 10^8^ (± 3.3 × 10^6^) cells mL^−1^ and 1.8 x 10^9^ (± 1.1 × 10^8^) cells mL^−1^. We evaluated different fixation methods and tested the sample stability. Stable abundance and fluorescence intensity were recorded up to 26 days during aerobic storage in PBS at 6 °C. The discrimination of the whole microbial community from the ubiquitous particle noise was facilitated by SYBR Green I staining and enabled calculation of relative abundances of methanogenic archaea of up to 9.64 ± 0.23% in the MEC and up to 4.43 ± 0.74% in the DS. The metaprofiling of the mcrA gene reinforced the results.

**Conclusions:**

The presented method allows for fast and reliable quantification of methanogenic archaea in microbial communities under authentic digester conditions and can thus be useful for process monitoring and control in biogas digesters.

**Electronic supplementary material:**

The online version of this article (10.1186/s12934-017-0793-7) contains supplementary material, which is available to authorized users.

## Background

In the last decades significant legislative and financial efforts towards a cyclic, more sustainable economic system were made. Focusing on the provision of energy and commodities from renewable resources, this has led to massive advancement and implementation of technologies like the anaerobic digestion of biomass to methane. Most anaerobic digesters are operated far from their theoretical volumetric productivity optima because higher loading rates significantly increase the probability of process failure due to acidification. To safely enhance the reactor performance, retain process stability and enable flexible, demand-driven biogas production even with variable substrates, a distinctive lack of low latency process control has to be overcome. The final, methane-generating step, catalyzed by methanogenic archaea has been identified as the bottleneck of the multi-stage anaerobic digestion process. A fast, inexpensive, and reliable way of quantifying abundance and activity of these microorganisms in typical reactor digestate would greatly improve process monitoring, enable control in biogas digesters and help optimizing the productivity of anaerobic digestion processes.

DNA sequence-based methods most frequently used today either rely on 16S rDNA specific for the most abundant subgroups of methanogenic archaea [1, 2], metagenomics analysis [3], or the functional *mcrA* gene coding for the α-subunit of the methyl coenzyme M reductase [4–6]. Fingerprinting methods, such as T-RFLP [7] provide qualitative abundance information, while single cell-labeling by fluorescent in situ hybridization (FISH) [8] or qPCR methods [9] can be employed for quantification. Some protocols aim to compute methanogenic activity by comparing qRT-PCR results with model-generated reference abundances [10]. The methyl coenzyme M reductase can additionally be quantified both on mRNA and protein level to provide activity information [10, 11]. Due to laborious protocols and in some cases elaborate data processing, none of the mentioned methods can be sustained as a routine measurement method for high frequency sampling without investing substantial money, time and workforce. The steering of running processes based on results of these methods is thus problematic.

Instead, methanogenic archaea can also be identified by their intrinsic fluorescent cofactor F_420_ (8-hydroxy-5-deazaflavin). Fluorescence microscopy based on the cofactor F_420_ was used as a direct, non-destructive and cost-efficient approach for the identification and quantification of methanogenic archaea in microbial communities [12, 13]. The cofactor was first described in 1972 [14] and displays a distinct blue fluorescence with an excitation maximum at 420 nm in its oxidized state. In contrast, the methanogenic cofactor F_430_ that displays a similar absorption spectrum and is build up by a recently uncovered synthesis pathway [15] shows no autofluorescence. The cofactor F_420_ is an essential hydride carrier in hydrogenotrophic methane synthesis [16–18]. The reduced cofactor F_420_ supplies reduction equivalents for the stepwise covalent binding of the second and third hydrogen atom in methanogenesis (CH–CH_2_–CH_3_) and is oxidized during the process. The quantification of cofactor F_420_ in pure culture extracts has been realized using either HPLC [19, 20] or assays with the ADP-linked hydrogenase system of *Methanobacterium bryantii M.o.H* [21]. The concentrations measured with either technique varied from 120 to 410 mg kg^−1^ cell mass (wet) for hydrogenotrophic methanogenic archaea but were considerably lower for organisms conducting non-hydrogenotrophic methanogenesis like *Methanosarcina* sp. (16 mg kg^−1^) [19, 21].

In this study we use the autofluorescent properties of the cofactor F_420_ for the fast, flow cytometric quantification of methanogenic archaea in biotechnologically relevant microbial communities. Flow cytometry is well tested for automated online analyses such as routine drinking water monitoring [22–24], anaerobic lab-system analysis [25], and full-scale digester monitoring [26]. It has also been widely used for analysis of autofluorescent microorganisms, even in mobile applications and open water environments [27–31].

We propose the use of a 405 nm laser to excite the cofactor F_420_. The study tested this excitation source for precise determination of abundance and autofluorescence intensity of F_420_ fluorescent cells in a variety of methane-producing microbial communities. These include a methanogenic enrichment culture and several communities from continuously operated digesters fed with industrial grade renewable or lab-designed substrates. We present a method including quality control steps for a reliable application in biogas reactor environments. The procedure was verified by MiSeq sequencing of flow cytometrically sorted subcommunities and T-RFLP analysis of whole samples.

## Methods

### Cultivation of microbial communities

A methanogenic enrichment culture (MEC) originating from a continuous stirred tank reactor [32] was obtained from the strain collection of the Helmholtz Centre for Environmental Research-UFZ Leipzig. The culture was kept under strict anaerobic conditions at 37 °C in 100-mL serum bottles filled with 50 mL-DSM 120 medium, containing a total of 4 g L^−1^ complex substrate usable as a carbon and energy source (Additional file 1: S1). Two percent (v/v) of the MEC was transferred every 4 weeks into new medium with an initial headspace atmosphere of 80% N_2_ and 20% CO_2_.

A second microbial community was acquired from the second stage of a two stage reactor system (Bräutigam Kunststoffsysteme GmbH, Mohlsdorf, Germany) of two 15-L continuous stirred tank reactors with 12 L working volume each. Material from this source will be referred to as digester sample (DS). The methanogenic stage was fed with 400 mL of acids and corn silage residues, containing a total of 66.58 g L^−1^ organic dry mass (composition in Additional file 1: S1) every 24 h. This substrate was produced in the preceding acidogenic reactor, fed with corn silage (from a farm in the nearby municipality of Neichen, Germany). The methanogenic digester was run at 38 °C (± 1 °C) with an organic loading rate (OLR) of 2 g L^−1^ day^−1^ and a hydraulic retention time (HRT) of 30 days. The S-shape agitator was propelled to 75 rpm by an overhead stirrer (RZR2102, Heidolph Instruments, Schwabach, Germany). Prior to sampling, the digester was operated in steady state for 4 retention times with a FOS/TAC value below 0.2, pH between 7.8 and 8, electrical conductivity between 18.0 and 20.7 mS m^−1^ and ammonia nitrogen concentrations between 1.2 and 1.4 g L^−1^ (digestate composition in Additional file 1: S1).

For the community screening test, digestate samples from eight different lab scale biogas reactors were obtained. The reactor and stirrer layout matched the digester sampled for the DS substrate input. The respective process parameters are given in Table 1 (see below). In short, the digesters were fed with disintegrated straw, whole-plant rye silage, corn silage, chicken manure, common duckweed, *Elodea nuttallii* and synthetic organic acids with an OLR between 1 and 4.65 g L^−1^ day^−1^ and an HRT between 8 and 285 days.

**Table 1:** Six digesters were screened cytometrically

Digester	Substrate	Process parameters				F420+	
		HRT [day]	OLR [g L^−1^ day^−1^]	Temp. [°C]	Stirring [min^−1^]	Cell number [mL^−1^] (± standard dev.)	Autofluorescence intensity (± standard dev.)
A	Disintegrated straw	60	2.5	40	100	1.24 × 10^9^ (± 4.98 × 10^8^)	33.57 (± 0.85)
B	Whole-plant rye silage	40	2.5	40	200	4.50 × 10^8^ (± 6.85 × 10^6^)	40.20 (± 2.31)
C	Corn silage	150	2.5	40	100	3.60 × 10^9^ (± 1.46 × 10^8^)	21.90 (± 0.56)
D	Chicken manure	60	2	30	100	2.40 × 10^9^ (± 2.04 × 10^8^)	38.13 (± 5.89)
E	Chicken manure	60	2	39	100	9.14 × 10^8^ (± 4.66 × 10^7^)	35.70 (± 3.22)
F	Common duckweed	40	1	40	70	6.17 × 10^7^ (± 2.42 × 10^6^)	33.03 (± 2.34)
G	*Elodea nuttallii*	285	2.7	37	160	1.02 × 10^9^ (± 1.80 × 10^8^)	26.37 (± 0.50)
H	Synthetic organic acids	8	4.65	37	50	2.23 × 10^9^ (± 8.60 × 10^7^)	48.73 (± 0.49)

The screened digesters differed in substrate input and their main process parameters. Cell numbers and mean intensities of the autofluorescent subcommunities F420+ are given with standard deviations

### Sampling, sample treatment and storage

Two-hundred µL aliquots were taken from the MEC serum bottles and of the digesters’ daily waste streams with a sterile, nitrogen-rinsed syringe and a clipped 1-mL pipette tip, respectively. After the transfer to 2-mL Eppendorf tubes (Eppendorf AG, Hamburg, Germany), a washing step (10 min, 4000 g, 10 °C) in 1.5 mL PBS buffer (1.8 g L^−1^ Na_2_HPO_4_, 0.223 g L^−1^ NaH_2_PO_4_, 8.5 g L^−1^ NaCl, pH 7.2) was performed. The pellet was resuspended in 1 mL PBS, filtered using 50-µm CellTrics^®^ (Sysmex Corporation, Kobe, Japan) and stored at 6 °C in the dark until measurement and/or cell sorting. Sample preparation and measurement for flow cytometry was done under aerobic conditions. All solutions used for cell treatment were cleaned of any particles using 0.2-µm syringe filters (Eppendorf AG).

### Cellular nucleic acid staining

SYBR Green I staining of DNA was applied to stain all cells in a sample. The staining was performed in 800-µL batches containing 5 µL sample solution, 735 µL PBS, 40 µL ethanol, and 20 µL 20× SYBR-Green I solution (ThermoFisher Scientific, Waltham, Massachusetts, USA). The samples were stained at least 3 h.

DAPI staining was applied according to [33] to provide high resolution cytometric community fingerprints. The samples were diluted to an optical density of 0.035 at 700 nm, incubated 20 min in 4.1 mmol L^−1^ Tween 20 and 0.11 mol L^−1^ citric acid and afterwards stained in 0.24 µmol L^−1^ DAPI (Sigma Aldrich, St. Louis, Missouri, USA) solution.

### Fluorescence microscopy

An Axio Scope.A1 fluorescence microscope (Carl Zeiss Microscopy GmbH, Jena, Germany) equipped with an Illuminator HXP 120 V, a plan-apochromat 100×/1.40 Oil DIC M27 objective, an Axiocam MRm camera and Axiovision software version 4.83 SP3 was used to visualize the microbial communities. F_420_ autofluorescence was visualized with a 395–440 nm excitation filter, a 475–495 nm emission filter, and a 460 nm beam splitter (Carl Zeiss). SYBR Green I fluorescence was visualized with a 475–495 nm excitation filter, a 515–565 nm emission filter and a 510 nm beam splitter (Carl Zeiss).

### Fluorescence spectroscopy

For analysis of the fluorescence properties the bulk samples were diluted to an optical density of 0.3 at 700 nm and measured in 3-mL quartz cuvettes with an F4500 fluorescence spectrophotometer (Hitachi, Chiyoda, Japan). The 3D fluorescence scans covered excitation and emission spectra from 330 to 600 nm in 5-nm steps, respectively.

### Flow cytometry

Cytometric measurements were performed with a BD Influx v7 Sorter USB, (Becton, Dickinson and Company, Franklin Lakes, USA) equipped with a blue 488 nm Sapphire OPSL (400 mW, Coherent, Santa Clara, USA), a violet 405 nm 56CRH OPSL (100 mW, Melles Griot, Carlsbad, USA) and a 355 nm UV Genesis OPSL (100 mW, Coherent).

The 488 nm laser was used for analysis of forward scatter (FSC, 488/10), side scatter (SSC, trigger signal, 488/10), and the SYBR Green I fluorescence (530/40), while the 405 nm laser excited the F_420_ fluorescence in methanogenic archaea (460/50) and the 355 nm laser excited the DAPI fluorescence (460/50). Light was detected by Hamamatsu R3896 PMTs in C6270 sockets (Hamamatsu, 211 Hamamatsu City, Japan). The fluidic system was run at 33 psi (2.275 bar) with sample overpressure at 0.5 psi and a 70-µm nozzle. The sheath fluid consisted of FACSFlow buffer (BD) diluted 1:2 with 0.1 µm filtrated Millipore water. For calibration of the cytometric set up in the linear range, 1 µm blue fluorescent FluoSpheres F-8815 Molecular Probes (Eugene, Oregon, USA) and 2 µm yellow-green fluorescent FluoSpheres F8827 (ThermoFisher Scientific, Waltham, Massachusetts, USA) were used. For calibration of the log range 0.5-µm UV Fluoresbrite Microspheres 18339 (Polysciences, Warrington, USA) were used. For cell analysis 1-µm blue fluorescent FluoSpheres F-8815 were added to every sample as control beads to ensure measurement stability and therefore allow comparison between samples. Samples were analyzed at a speed of 6000 events s^−1^. Cells of five subcommunities per sample were sorted, four subsamples at a time, using the most accurate sort mode “1.0 drop Pure” and with an event count rate of 15,000 s^−1^. 500,000 cells per sample were acquired. The cells were collected in a pellet after supernatant removal in two subsequent centrifugation steps at 20,000*g*, 6 °C for 25 min and 5 min, respectively. The cell pellets were stored at − 20 °C. Cytometric data were evaluated using FlowJo v10.0.8r1 with the Engine v3.04910 (FlowJo, LLC, Ashland, USA) and flowCyBar [34] using the R package flowCyBar (https://www.bioconductor.org/packages/release/bioc/html/flowCyBar.html).

### Cell counting

Cell counting was performed by adding 1-µm yellow-green fluorescent FluoSpheres beads F13081 with a microscopically determined concentration to every sample (in triplicates). The measured sample volume needs to be related to cell numbers which are recorded in a preset gate to calculate cell numbers per mL sample. The gating strategy introduced as a base for this calculation is displayed in Additional file 1: Figure S4. While the F_420_ autofluorescent subcommunity (F420+) cell count does not need any staining, the total cell number of the whole community needs to be differentiated from debris by SYBR Green I staining. The location and size of the SYBR Green I gate marks also the location of those cells in the SSC/FSC 2D-plot which were subsequently chosen for all cell number countings in unstained samples. The difference of this number and the autofluorescent F420+ cells provides the number of non-fluorescent cells (F420−).

### Sequencing

Whole communities and both sorted F420+ and F420− subcommunities of one MEC and two DS aliquots (DS1 and DS2) were examined by MiSeq sequencing. The DS aliquots were taken from the steady state digester within an interval of 2 weeks. DNA extraction was performed according to [33] using 70 µL of 10% Chelex 100 solution (Biorad, Hercules California, USA) for 500,000 cells. The extracted DNA was stored at − 20 °C. The library was created with a two-step PCR comprising 20 cycles with the mother primers (MLF and MLR) [4] and 10 cycles with the barcoded primers. The sequencing data were generated by an Illumina MiSeq sequencer with the v3 kit, 2 × 300 bp, 600 cycles option (Illumina, San Diego, California, USA) by Fasteris (Fasteris SA, Plan-les-Ouates, Switzerland). The dataset was processed and evaluated using Mothur [35] and UCHIME [36] and visualized with the ggplot2 package in R [37]. Details concerning DNA extraction, PCR, sequencing and the data processing steps are explained in Additional file 1: S9.

### T-RFLP

The screening of methanogenic community composition in the digesters was performed by terminal restriction fragment length polymorphism (T-RFLP) analysis according to standard procedures [7]. In short, DNA was extracted with a NucleoSpin Soil kit (Macherey–Nagel, Düren, Germany) using the lysis buffers SL 1 and SX and the FastPrep lysis 4.0 for 20 s. PCR amplification of the *mcrA* gene was performed with the mlas (5 pmol μL^−1^, 5-GGTGGTGTMGGDTTCACMCARTA-3) [2], and *mcrA*-rev (5 pmol μL^−1^, 5′-CGTTCATBGCGTAGTTVGGRTAGT-3′) [4] primer pair. Restriction digestion was performed with the endonucleases *Mwo*l and *Hae*III (New England Biolabs) [38] and data analysis was performed with the ABI PRISM Genetic Analyzer 3130xl (Applied Biosystems, Darmstadt, Germany). Species allocation of the terminal restriction fragments (T-RFs) was conducted using a database for biogas digesters [7].

## Results

### F_420_ fluorescence in methanogenic archaea

**Fig. 1:**
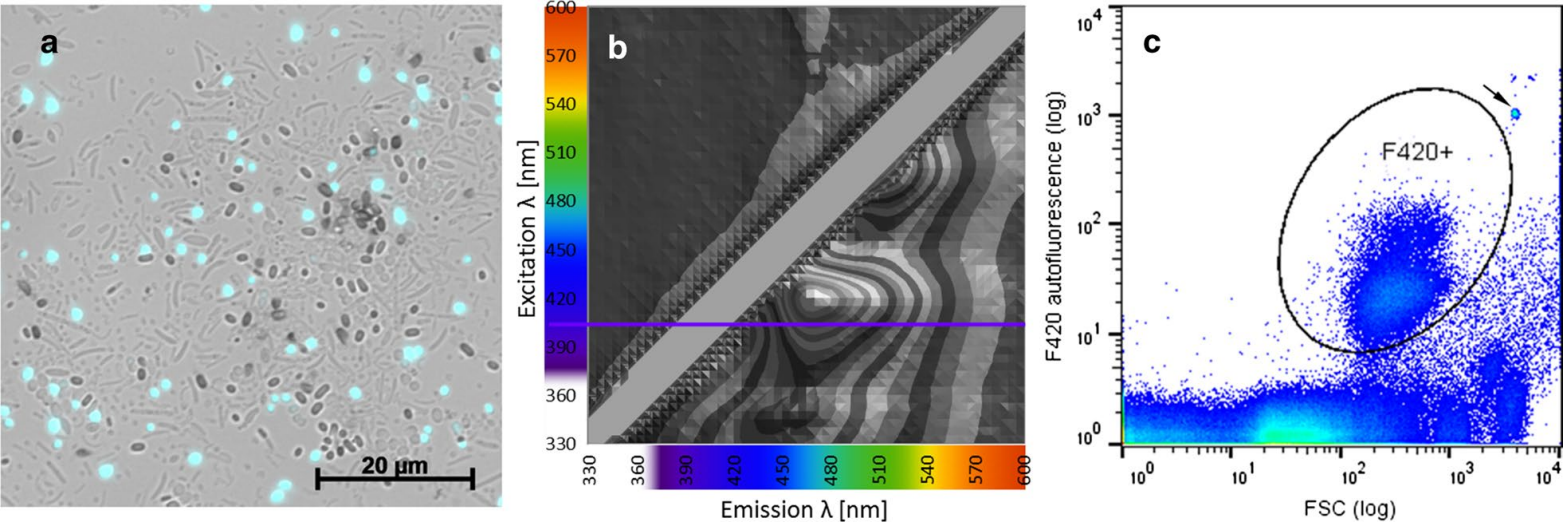
Visualization of the cofactor F_420_ autofluorescence in the methanogenic enrichment culture with **a** fluorescence microscopy, **b** fluorescence spectroscopy (405 nm excitation is marked with a purple line) and **c** flow cytometry. The gate F420+ is indicated. The arrow marks the added control beads (details in “Methods”)

**Fig. 2:**
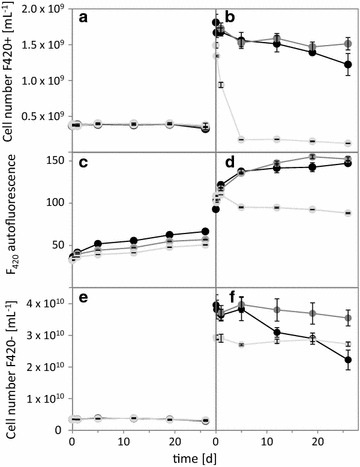
Sample stability of the microbial community of the methanogenic enrichment culture (**a**, **c**, **e**) and the digester sample (**b**, **d**, **f**). The material was stored in PBS at 22 °C (room temperature, black circle), 6 °C (dark grey circle) and (light grey circle) 0 °C (on ice, light grey circle). **a**, **b** Absolute numbers of autofluorescent cells (gate F420+). **c**, **d** Mean F_420_ fluorescence intensity of cells in gate F420+. **e**, **f** Absolute cell numbers of non-autofluorescent cells (gate F420−). The error bars represent the standard deviation of the three replicates

Using fluorescence spectroscopy and a test culture of enriched methanogens (MEC) we recorded the characteristic excitation (max. 420 ± 2.5 nm) and emission spectra (max. 470 ± 2.5 nm) of cofactor F_420_ (Fig. 1b). By iterating along these values we tested different flow cytometer configurations to optimize for both, F_420_ autofluorescence resolution and mean intensity, in order to discriminate F_420_ autofluorescent from non-autofluorescent subcommunities of the MEC. Subsequently, the flow cytometric identification of methanogenic archaea was achieved using a 405 nm laser and a 460/50 nm emission filter. The violet light excites the cofactor F_420_, which is ubiquitous in methanogenic archaea and unique to this taxon at the identified concentrations [19] (Fig. 1c). With the 405 nm laser we found adequate subcommunity discrimination while the 355 nm UV laser and respective emission filter set did not provide sufficient discrimination (Additional file 1: Figure S2). The MEC and an additional digester sample (DS) were compared to a control community that did not contain methanogenic archaea and was obtained from an acidogenic digester without any CH_4_ production capacities (Additional file 1: Figure S3). The non-methanogenic control clearly lacked the F_420_ autofluorescent subcommunity (F420+) visible in the CH_4_ producing communities. To enable algorithm-based quantification of the F_420_ autofluorescent subcommunity we set a marker gate for the subpopulation F420+ (Fig. 1c). The observed cell numbers in F420+ were about one order of magnitude lower in the MEC than those in the DS (3.7 × 10^8^ ± 3.3 × 10^6^ mL^−1^ in the MEC, 1.8 × 10^9^ ± 1.1 × 10^8^ mL^−1^ in the DS, Fig. 2).

### Sample stability

Flow cytometric online/on site measurement routines for biogas digesters have not been established and digestate needs to be sampled, transported and stored in order to analyze the contained microbial community. To identify a reliable storage protocol that causes minimal technical biases to the recorded community structure and fluorescence intensity, five storage protocols were tested with DS over a 3 day period: (1) storage in PBS at 6 °C, (2) fixation by 2% formaldehyde for 30 min and subsequent storage in PBS at 6 °C, (3) vacuum drying for 40 min at 35 °C and 550 g and storage of the pellets at 6 °C, (4) storage in 15% glycerol/PBS at 6 °C and (5) storage in 15% glycerol/PBS at − 20 °C. The flow cytometric analysis of each sample was compared to the freshly measured control (Additional file 1: S5). Two of the tested protocols (1 and 2) showed good conserving properties. We favored protocol (1) for all subsequent experiments because sample handling was easy and toxic chemicals were avoided. Whereas protocols (3) and (4) worked similarly well, protocol (5) induced a substantial loss of F_420_ autofluorescent cells and autofluorescence intensity and altered the subcommunity structure in comparison to the fresh control.

Subsequently, we tested the long term sample stability of protocol (1) and assessed the most beneficial storage temperatures. Both MEC and DS were monitored for this purpose at three different temperatures over 26 days (22, 6, 0 °C, Fig. 2, details in Additional file 1: S6). High mean fluorescence intensities (FI_mean_) of autofluorescent F420+ cells were detectable over the whole test period. Even a slight increase in FI_mean_ was apparent for all samples except for DS at 0 °C. Stable F420+ and F420− cell abundances were detected in the MEC for all temperatures, but the DS showed a fast decrease at 0 °C, and gradual decreases at 22 °C. Storage at 6 °C showed comparably stable F420+ and F420− values of the MEC and DS samples for the whole test period (abundance changes: MEC F420+ − 4%, DS F420+ − 16%, MEC F420− − 10%, DS F420− − 10%) and was therefore chosen as the standard procedure.

### Community analysis through nucleic acid staining

So far, autofluorescent cells (F420+) could clearly be discriminated from other cells and quantified by the developed workflow. But non-fluorescent microorganisms (F420−) remained included in the range of instrumental noise, as well as abiotic particles and plant debris, which are ubiquitous in biogas digestate. Therefore, the all-cell SYBR Green I nucleic acid stain was applied, since this dye has been comprehensively tested for applications in online flow cytometry systems [24]. This stain allowed discrimination of cells from debris and thus cell counting of F420− cells which were present on average at 3.5 × 10^9^ ± 3.2 × 10^7^ cells mL^−1^ in MEC and 4.0 × 10^10^ ± 3.3 × 10^9^ cells mL^−1^ in DS. F420− cell numbers were approximately one order of magnitude higher than the corresponding autofluorescent F420+ cell numbers (9.64 ± 0.23% of the MEC, 4.43 ± 0.74% of the DS).

**Fig. 3:**
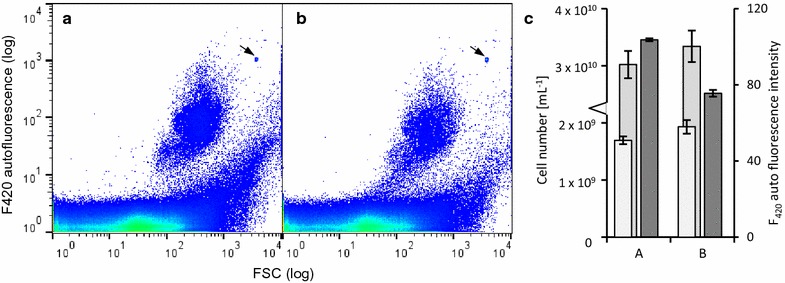
Influence of nucleic acid staining on F_420_ fluorescence. **a** Unstained digester sample after 3 h as a control. **b** The same sample after 3 h of SYBR Green I staining. **c** Cell numbers of the subcommunities F420+ (white bar), F420− (light grey bar) and autofluorescence intensity (dark grey bar) of the subcommunities F420+ are indicated with the respective standard deviations. Samples were gated according to Additional file 1: Figure S4. Values are given in Additional file 1: S7. The arrow marks the added control beads

Since the use of fluorophores can quench autofluorescent properties, as is commonly known for multi-fluorescent labeling approaches [39], we were specifically interested in testing the FI_mean_ autofluorescent values after SYBR Green I staining. We indeed observed a drop in autofluorescent FI_mean_ for the F420+ subcommunities in both MEC and DS (FI_mean_ reduction: MEC − 31%, Additional file 1: S7; DS − 25%, Fig. 3). Nonetheless, the F420+ subcommunity was still clearly discriminated from the non-fluorescent cells and the respective cell numbers hardly changed (4.8 × 10^8^ ± 1.31 × 10^7^ cells mL^−1^ unstained, 5.6 × 10^8^ ± 1.64 × 10^7^ cells mL^−1^ stained).

**Fig. 4:**
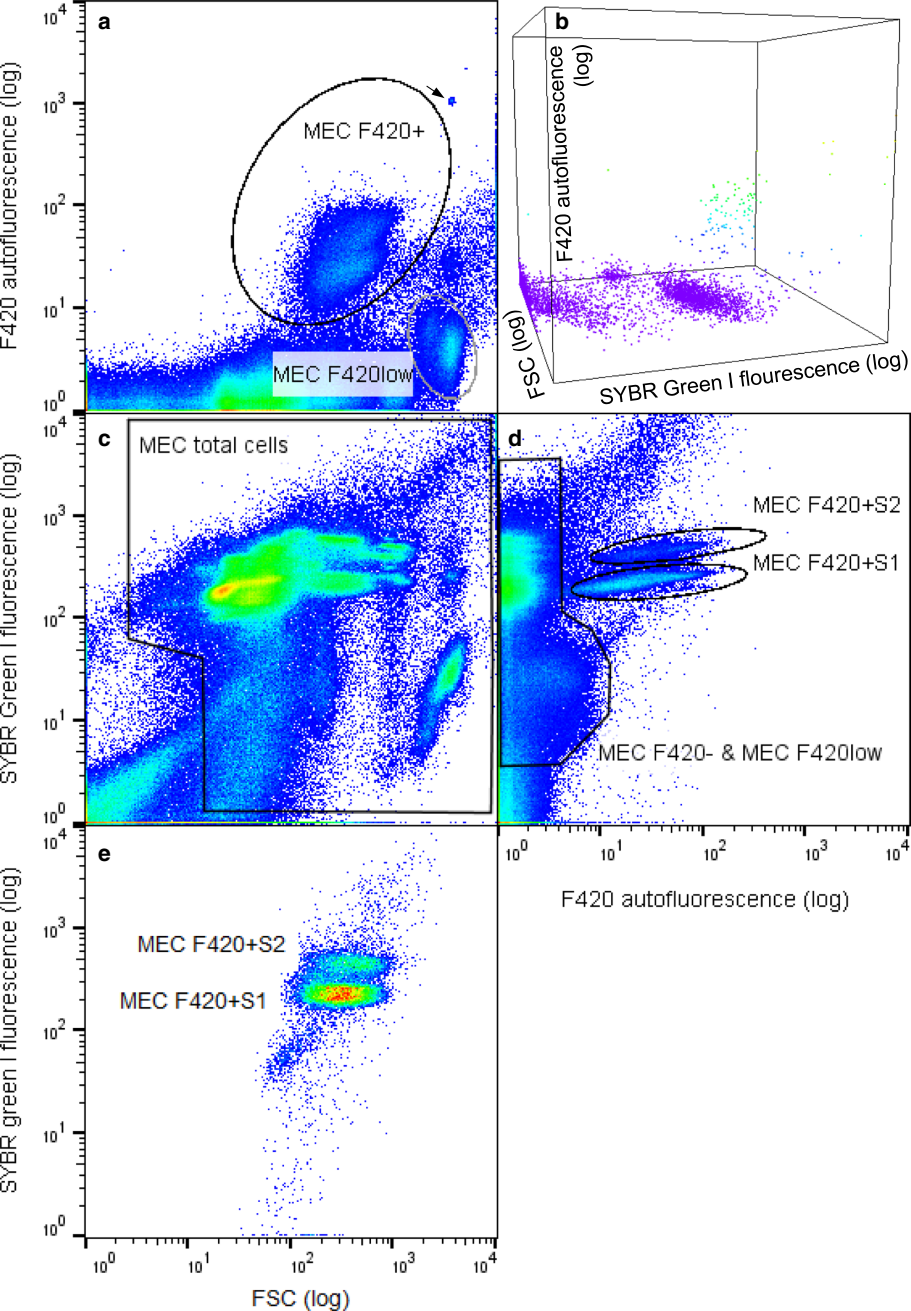
Flow cytometric analysis of a methanogenic enrichment culture (MEC) with sort gates in black (**a** unstained, **b**–**e** stained). **a** FSC vs. F420+. Subcommunities with high autofluorescent (MEC F420+) and low autofluorescent properties (MEC F420low) can be detected. **b** 3D visualization of FSC vs. F420+ vs. SYBR Green I. **c** FSC vs. SYBR Green I plot of the total microbial community. **d** F420+ vs. SYBR Green I plot used for discrimination of high (MEC F420 + S1 and MEC F420 + S2) as well as low and non-autofluorescent subcommunities (MEC F420− and MEC MF420low. **e** Position of subcommunities MEC F420 + S1 and MEC F420 + S2 in a FSC vs. SYBR Green I plot. The arrow marks the control beads

Besides cell number determination a range of further routines, such as cytometric fingerprinting in the FSC vs. SYBR Green I plot (Fig. 4c) similar to DAPI based protocols [33] are applicable. Established DAPI protocols are not combinable with the detection of methanogenic archaea because of emission spectrum overlaps. Testing the MEC, additional subcommunities emerged that were not clearly discriminable by autofluorescence (Fig. 4a) or SYBR Green I alone (Fig. 4c) but by the combination of both (Fig. 4d). These subcommunities were named MEC F420 + S1 and MEC F420 + S2 (Fig. 4e). The DS also showed enhanced discrimination of autofluorescent subcommunities (Additional file 1: S8). Apparently, the staining procedure did not only allow quantification of autofluorescent (F420+) vs. non-fluorescent microorganisms (F420−), but also enabled the discrimination of autofluorescent subcommunities which were not distinguishable before.

### Verification of cytometric data by MiSeq sequencing

**Fig. 5:**
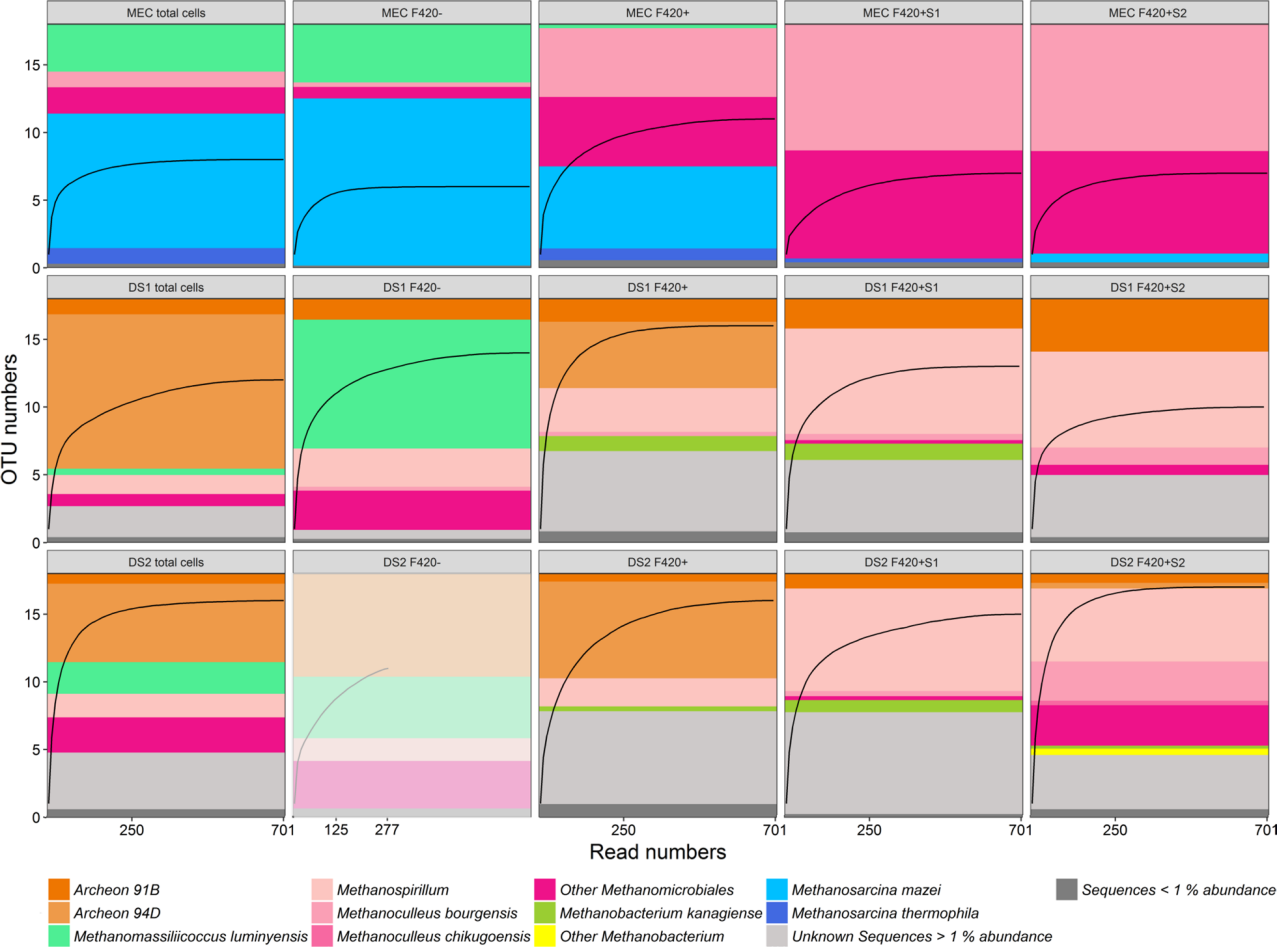
Rarefaction curves (black line), relative abundances [%] and taxonomical assignment of *mcrA* reads (colored bars) of the methanogenic enrichment culture (MEC total cells) and the digester samples (DS1, DS2 total cells) as well as for sorted subsamples (F420−, F420+, F420 + S1, F420 + S2) examined by Illumina^®^ Sequencing. The read number was normalized to 701 per subsample. DS2 F420− only represented 201 reads. Only OTUs with over 1% abundance are shown. The rarefaction curves are displayed with a 0.1% abundance threshold

Using flow cytometry we assigned parts of the analyzed microbial communities to the functional group of methane-producing cells based on F420+ autofluorescence and SYBR Green I discrimination. To verify this assignment with an independent method and to test the performance of the introduced staining protocol, we analyzed the phylogenetic compositions of 12 subsamples after cell sorting by Illumina MiSeq sequencing. The phylogenetic affiliation was based on *mcrA* sequence detection and reads assignment was performed with an established data base [40]. The MEC and two digester samples (DS1 and DS2) were used as controls for total communities (Fig. 5). The subsamples were F420−, F420+, F420 + S1, and F420 + S2 for all three tested communities (Fig. 5). A total of 71.099 overlapped *mcrA* amplicon reads were obtained after cleaning, quality control steps and merging of the forward and reverse sequences (Additional file 1: S9). Fifteen subsamples with 277–13,402 sequences per subsample and an average of 4740 sequences per subsample were normalized with a threshold of 701 sequences per subsample. This threshold excluded the lowest read number subsample (DS2 F420−) to enhance the overall statistical validity. The excluded subsample yielded sufficient sequences for OTU analysis (277) but should not be directly related to the others (opaque in Fig. 5). The two lowest read numbers were found in the non-autofluorescent F420− subcommunities DS1 F420− (701) and DS2 F420− (277). The final analyzed data set contained 9814 sequences that were classified and clustered into 28 OTUs with more than 0.1% abundance (Fig. 5).

Cytometric analysis revealed a MEC community structure that was different from the two comparable DS communities. This relation was also observed on the OTU level. The MEC showed lower OTU and read numbers in comparison to the DS samples (MEC 12 OTUs, DS1 25 OTUs, DS2 26 OTUs, 0.1% threshold, Additional file 1: S9). In addition, the taxonomic affiliation and relative abundance of the OTUs were much more similar between the DS samples. Furthermore, 99.2% of the overall MEC reads could be affiliated, while only 78.1% of the DS1 and 67.6% of the DS2 reads belonged to taxonomically described OTUs.

Illumina sequencing revealed that the OTU compositions of both the total MEC as well as the total DS subsamples (controls) corresponded to the respective combined F420− and F420+ subsamples. Only two low abundance OTUs were additionally identified in the F420+ subsamples (*Methanobacterium kanagiense:* 2% in DS2 F420+, 6.1% in DS1 F420+, *Methanoculleus bourgensis*: 1.7% in DS1 F420+).

For all three sample sets the OTU distribution between F420− and F420+ varied substantially and suggests a relation between state of metabolism and gate allocation. *Methanosarcina mazei* (68.6% in MEC) and *Methanomassiliicoccus luminyensis* (23.8% in MEC and 52.9% in DS1), both capable of non-hydrogenotrophic methanogenesis, were dominant in F420− subsamples. Two *Methanomicrobiales* sp. (MEC 54.6%) that favor the hydrogenotrophic pathway and the *Archeon 94D* (DS1 27.2%, DS2 39.7%) were the most abundant OTUs in F420+.

When comparing OTU compositions between F420+ and the combined F420 + S1 and F420 + S2, frequently similar OTU types and abundances were detectable, although a few individual OTUs were no longer present. The MEC contained *Methanoculleus bourgensis* and other *Methanomicrobiales* OTUs, which were represented at the same ratio in all three subsamples, while the facultative non-hydrogenotrophic *Methanosarcina* sp. were less abundant in F420 + S1 (− 96%) and F420 + S2 (− 87%). In the DS all three subsamples comprised the *Archaeon 91B, Methanospirillum* and small abundances of *Methanobacterium kanagiense*. *Methanoculleus bourgensi* and other *Methanomicrobiales* and *Methanobacterium* OTUs were also identified in the more specific F420 + S1 F420 + S2 gates, while only the *Archaeon 94D* was less abundant.

It became apparent, that the phylogenetic distinction and comparison of the MEC and the two similar DS samples further verified the good resolution of the cytometric analysis. The sorting procedure permitted taxonomic in-depth analysis and revealed even higher numbers of OTUs in comparison to total communities.

### Digester screening

**Fig. 6:**
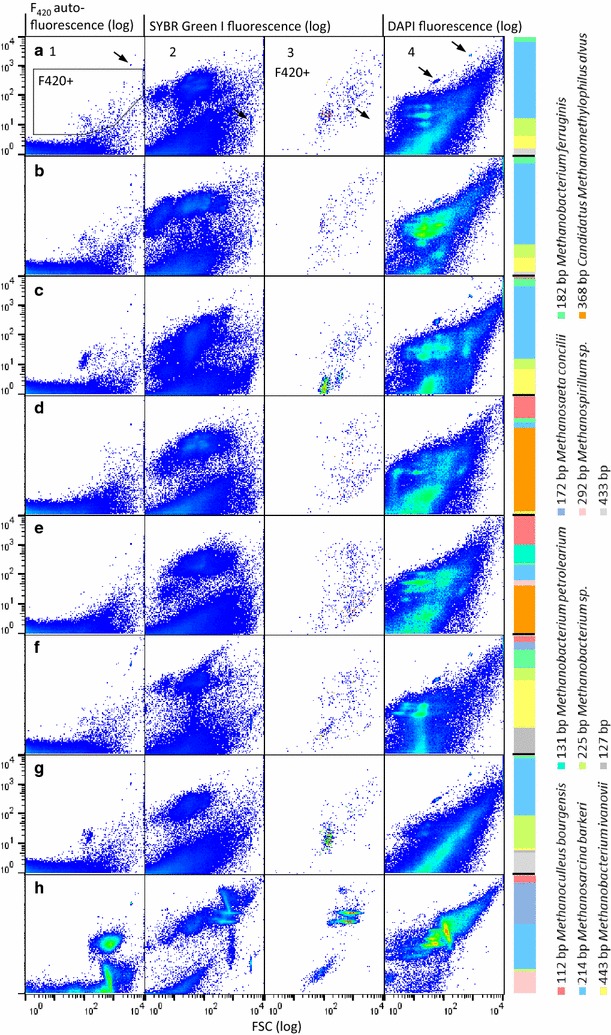
Community analysis of six digesters **a**–**h**. Column **1**: Flow cytometric measurement of the unstained samples with the respective autofluorescent subcommunities F420+. Column **2**: The total SYBR Green I stained digester communities. Column **3**: SYBR Green stained subcommunity F420+. Column **4**: the total DAPI stained digester communities. A *mcrA* targeted T-RFLP analysis of methanogenic archaea in the fresh samples is shown for each digester. Unidentified T-RFs are indicated in grey. The digesters were fed with **a** disintegrated straw, **b** whole plant rye silage, **c** corn silage, **d**, **e** chicken manure, **f** common duckweed, **g** *Elodea nuttallii* and **h** synthetic organic acids. 1,000,000 total events were recorded for unstained samples **a**–**g** while 200,000 events were recorded for unstained sample **h**; 100,000 cell events were recorded in the SYBR Green I stained samples; 200,000 cell events were recorded in the DAPI stained samples. The black arrow marks the control beads (details in “Methods”)

To demonstrate the general applicability of the presented procedure to different digestate textures and microbial communities, a selection of continuous stirred tank reactors (A–H) was screened. The digesters were identical in design and operated with different process parameters and industry grade or artificial substrates (Table 1). The digestates were flow cytometrically analyzed using unstained and SYBR Green I stained samples, as well as DAPI staining for cytometric fingerprinting. A *mcrA* targeted T-RFLP analysis was performed for every sample to verify observed differences in the methanogenic community structure. Figure 6 represents autofluorescent F420+ subcommunities, the SYBR Green I and the DAPI fingerprints, as well as SYBR Green I stained F420+ subcommunities. The cell abundance recorded in the subcommunity F420+ (gate F420+, Fig. 6a1) ranged from 6.17 × 10^7^ cells mL^−1^ (± 2.42 × 10^6^) in digester F to 3.60 × 10^9^ cells mL^−1^ ± 1.46 × 10^8^ in digester C. The mean autofluorescence intensities of subcommunity F420+ ranged from 21.90 (± 0.56) in digester C to 48.73 (± 0.49) in digester F. The highest particle load was found in digester G probably introduced by fractured plant cells of the *Elodea nuttallii* feed, visible in the 2D-plots G2 and G4. However, the 2D-plot G1 showed a clearly discriminated autofluorescent cell cluster which allowed cell number (1.02 × 10^9^ cells mL^−1^ ± 1.80 × 10^8^) and fluorescence intensity determinations (26.37 ± 0.50). Samples from digester H featured a high F420+ cell number (2.23 × 10^9^ mL^−1^ ± 8.60 × 10^7^) and the most segregated subcommunities. The cultivation on synthetic organic acids may have selectively enriched methanogenic archaea, whereas the very low particle load in the sample supported a high resolution measurement.

Using *Mwo* l for restriction digestion, 18 T-RFs with an abundance of more than 1% ranging from 51 to 443 bp were found after processing of all samples. Figure 6 shows T-RFs with at least 10% abundance in at least one digester (1% threshold fingerprint in Additional file 1: Figure S10). *Methanosarcina barkeri* was identified in all digesters with abundances between 67% (B) and 1% (F). *Methanosaeta concilii* was only present in digesters F (5%) and H (35%). The microbial communities in the digesters A, B and C show a similar T-RF distribution and were all fed with classical renewable substrates i.e. disintegrated straw, whole plant rye silage and corn silage, respectively. The two chicken manure fed digesters D and E displayed very similar SYBR Green I and DAPI fingerprints. They distinctly differed from other samples and contained only small autofluorescent subcommunities. T-RF data clearly confirm the flow cytometric analysis by indicating the unique and high abundance (D 67%, E 36%) of *Candidatus Methanomethylophilus alvus* in both samples. The digester screening confirmed that the developed workflow can be successfully applied to different digestates including the most common renewable substrate (i.e. corn silage [41]) in industrial/agricultural digesters.

## Discussion

The global microbial CH_4_ production is estimated to reach one billion tons annually. Methanogenic archaea produce CH_4_ in wetlands, rice fields, ruminant and termite digestive systems and have a decisive impact on the planet’s atmospheric carbon cycle [42]. At the same time, the industrial scale anaerobic digestion of biomass to CH_4_ plays a vital role in the future global energy mix. All methanogenic archaea capable of CO_2_ reduction contain the cofactor F_420_ as an integral part of the methanogenic pathway. In this study, F_420_ autofluorescence was tested as a universal marker for methanogenic archaea. Genes encoding for F_420_ biosynthesis enzymes were identified in 653 bacterial and 173 archaeal species [43]. Non-methanogenic but F_420_ containing microorganisms have reported F_420_ concentrations of about one fortieth of the concentrations in hydrogenotrophic methanogenic archaea [19], which is below detection limit of the developed protocol. For the methanogenic archaea, however, the F_420_ cofactor autofluorescence served successfully for cytometric cell counting, and we found between 6.17 × 10^7^ (± 2.42 × 10^6^) and 3.60 × 10^9^ (± 1.46 × 10^8^) mL^−1^ organisms in all investigated communities. The results are in accordance with previous qPCR studies that recorded 10^8^ to 10^10^ mL^−1^ methanogenic archaea in microbial communities of biogas digesters [44]. The number of methanogenic archaea is obviously dependent on reactor conditions. The recorded abundances in literature correlated positively with the OLRs (3–5 g L^−1^ day^−1^) and reaction volume related methane productivities (0.8–2 L_CH4_ L^−1^ day^−1^) of investigated digesters [44]. We could confirm this correlation. The high OLR (2.0 g L^−1^ day^−1^) and methane productivity (0.91 L_CH4_ L^−1^ day^−1^, Additional file 1: S2) of the two test digester samples (DS) showed a methanogenic abundance approximately one magnitude higher than the batch methanogenic enrichment culture (MEC) with very low cumulative OLR (0.143 g L^−1^ day^−1^) and volumetric methane productivity (0.029 L_CH4_ L^−1^ day^−1^). This trend was also confirmed for the exemplarily screened digesters. The lowest abundance of methanogenic archaea was recoded in digester F (6.17 × 10^7^ mL^−1^ ± 2.42 × 10^6^), which was operated with the lowest OLR (1 g L^−1^ day^−1^).

As stability of the F_420_ autofluorescence in methanogenic archaea is controversially discussed in the literature, the established protocol required a verification of its integrity after each step in the workflow. In the past the sole presence of F_420_ autofluorescence has been regarded as a marker of metabolic activity [13] while other studies relied on *mcrA* copy numbers to quantify methanogenic archaea [45] and relate these abundances to activity [44]. Generally, F_420_ autofluorescence can be affected by (I) shifts between the fluorescent oxidized F_420_ and non-fluorescent reduced F_420_H states of the cofactor [46], (II) membrane integrity of the fluorescent cells and (III) fluorescence quenching of the cofactor F_420_. Aerobic environments have been reported to increase the autofluorescence in methanogenic archaea because the non-fluorescent F_420_H, still abundant in the cells, is oxidized [46]. Nevertheless it needs to be kept in mind, that some methanogenic archaea are considered to be vulnerable to oxygen and may be damaged by its influence [12]. Our samples were generally treated and measured under oxic conditions, but we found largely stable abundances (− 4%) in the autofluorescent subcommunities (F420+) even after 26 days in PBS at 6 °C (Fig. 2 and Additional file 1: S7). Therefore, PBS and 6 °C were selected as standard conditions for cell storage. Other tested storage procedures were not as stable: one reactor sample (PBS, 0 °C) showed decreased F_420_ cell numbers after only a few days (− 93.15%, Fig. 2) and it can be assumed that part of the autofluorescent cells were destroyed (Additional file 1: S7). This may also apply to cell drying and two glycerol-based methods [47] which severely altered both cell integrity (analyzed by abundance reduction of F_420_ autofluorescent cells; − 66.8, − 46.8, − 76%), and fluorescence intensity (analyzed by mean FI reduction; − 78.1, − 7, − 38.2%) and, consequently, are also not recommended. Photobleaching can diminish the cofactor F_420_ autofluorescence intensity [14, 48, 49] and potentially impact microscopic counting. However, flow cytometry analyses only require exposure times of 0.75–1.5 µs per cell and will therefore not bias the quantification of methanogenic archaea.

Fluorescence quenching could also occur due to energy transfer to salts, cell components and other fluorophores upon collision (Dexter electron transfer, [50, 51]) or over short distances (Fluorescence resonance energy transfer, FRET, [52, 53]). We observed such phenomena to a minor degree when the methanogenic archaea were stained with SYBR Green I, which was used to differentiate cells from the background noise and to further segregate the autofluorescent community F420+ into F420 + S1 and F420 + S2. The nucleic acid dye led to a decrease of F420+ autofluorescence specifically in case of *Methanosarcina* sp. which emerged with high relative abundance in the MEC F420− subcommunity (68.6%, Fig. 5). *Methanosarcina* sp. was also missing in the sorted F420 + S1 and F420 + S2 cell subsamples of the MEC, while in the DS samples another phylotype was absent (*Archeon 94D*). The *Archeon 94D* does not seem to undergo this shift consistently, as it is not found in DS1F420− but contained in the very low read number subsample DS2F420−. But both OTUs were otherwise stably present in sorted non-SYBR Green I stained F420+ communities. However, differentiation from noise using SYBR Green I for methanogen cell counting was valuable since particles and plant debris are ubiquitous in biogas digesters [1] (Fig. 6). In our applications the number of debris events reached on average 36 and 18.4% of all events in DS and MEC, respectively. The SYBR Green I staining step is thus a trade-off between a good separation of cells from plant debris along with highly segregated cell clusters and the risk of losing species.

Even without quenching, the cofactor F_420_ autofluorescence intensity is different between methanogenic archaea. The cofactor F_420_ is an immanent part of the hydrogenotrophic pathway but some methanogenic archaea can utilize acetate or methanol that enter the pathway at the level of –CH_3_ and bypass the F_420_ dependent steps [54]. This can lead to active methanogenic archaea with reduced F_420_ concentrations that may remain undetected with both, microscopic [12] and flow cytometric analyses. We indeed found the methylotrophic *Methanomassiliicoccus luminyensis,* a representative of the human gut microbiome [55], with 53.0% in the DS1 F420− subsample. Likewise, another methylotrophic, *Candidatus Methanomethylophilus alvus* [56], was detected with as much as 67.3 and 35.8% of the total T-RFs in the chicken manure fed digesters D and E respectively, in which only little autofluorescence was detected.

Despite these exceptions corresponding to a distinct type of methanogens, the vast majority of overall *mcrA* specific sequences were recorded in the autofluorescent subsamples. More than 19 and 30 times the read numbers of the non-autofluorescent subsample F420− were detected in the respective F420+ subsamples in DS1 and DS2. And even more than 22 and 54 times the read numbers were detected in the combined subsamples F420 + S1 and F420 + S2. The autofluorescent subsamples contained almost exclusively hydrogenotrophic organisms like *Methanomicrobiales,* with high abundances of *Methanoculleus sp.* in the MEC (51.8% in F420 + S1, 52.1% in F420 + S2) and *Methanospirillum* in the two DS (DS1: 43.2% in F420 + S1, 39.3% in F420 + S2, DS2: 30.0% in F420 + S1, 42.1% in F420 + S2). Additionally, the DS contained obligate hydrogenotrophic *Methanobacteriales* like *Methanobacterium kanagiense*. Those taxa have been shown to dominate the microbial communities in industrial scale biogas plants for crop (co)-digestion [57–59]. Indeed, we found multiple *Methanobacterium* species in the crop-fed digesters A, B and C in the digester screening (Fig. 6). Furthermore, in the synthetic acid fed digester H a high abundance (3.60 × 10^9^ cells mL^−1^ ± 1.46 × 10^8^) F_420_+ subsample was recorded that might contain hydrogenotrophic *Methanosarcina barkeri* capable of using all four methanogenic pathways [60]. The related T-RF abundances were high (38.7%) while most of the remaining T-RFs were affiliated to the obligate acetoclastic *Methanosaeta concilii* (35.0%) with low cofactor F_420_ content [61].

Altogether, the combination of flow cytometric analysis, cell sorting and molecular biology tools facilitated a much more profound characterization of the investigated microbial communities than bulk sequencing approaches can offer. For instance, we consistently found more different OTUs in the combined F420−, F420+, F420 + S1, F420 + S2 subsamples (MEC: 12, DS1: 25, DS2: 26), than for the total samples (MEC: 8, DS1: 12, DS2: 16). Additionally it needs to be mentioned that the presented flow cytometric quantification protocol for methanogenic archaea can achieve acquisition frequencies of up to 6000 events s^−1^. A typical cytometrically analyzed digester sample includes 1,000,000 recorded events and can be measured in about 3 min. The short acquisition times and the possibility to use unstained samples for the quantification of methanogenic archaea allowed the establishment of a quasi-online workflow. Flow cytometers are well acknowledged for bioprocess control [62] and if small bench top flow cytometers are equipped with less expensive 405 nm lasers, cell number dynamics of methanogenic archaea can be easily tracked in digesters.

## Outlook

Ongoing technical advances are improving the availability of 420 nm lasers with even higher optical output power [63] for enhanced F_420_ fluorescence resolution. This can help to further spread the fast, cost-effective single cell monitoring of F_420_ fluorescence intensity and overcome the current limitations in discriminating non-hydrogenotrophic methanogenic archaea with lower cofactor F_420_ concentrations. This in turn might enable deeper insight into shifts between methanogenic pathways in complex microbial communities by changes in fluorescence intensity. The combination of a robust sample treatment protocol, online flow cytometry [23] and automated data processing (CHIC [64], CyBar [33]) supports the concept of a “community sensor” with faster response times than traditional abiotic process parameters can provide.

## Conclusions

Flow cytometric single cell analysis can be applied to gain information about structure and function of biogas producing microbial communities. Being the major bottleneck for process stability, methanogenic archaea are the subcommunity of major interest. These methanogenic archaea can be discriminated and quantified due to their distinctive fluorescence. We recorded methanogenic abundances in accordance with the published values that correlated with substrate availability. Whilst recording the given abundances, we could show the fast, easy and cost effective nature of the quasi online flow cytometric analysis of hydrogenotrophic methanogenic archaea. Some non-hydrogenotrophic methanogenic archaea containing much lower cofactor F_420_ concentrations were only visible with low fluorescence intensity. The SYBR Green I staining was successfully implemented to discriminate non F_420_ fluorescent cells from the particle noise and still allowed discrimination of the F_420_ fluorescent subcommunity. Cell sorting and sequencing verified the flow cytometry-based subcommunity allocations. The digester screening demonstrated the applicability of the presented method to various digestates.

## Additional file

**Additional file 1.** Additional information containing details about: **S1** the sample origin, **S2** the flow cytometer channel test, **S3** the comparison of F_420_ fluorescent communities and a non-methanogenic control community, **S4** the gating strategy used for the cell number determination, **S5** the storage protocol testing, **S6** the influence of sample storage on F_420_ fluorescence, **S7** the nucleic acid staining, **S8** the community analysis, **S9** the sequencing protocols and details and **S10** the digester screening.

## Abbreviations

F_420_: autofluorescent cofactor of the methanogenesis
*mcrA*: α-subunit of the methyl-coenzyme M reductase
MEC: methanogenic enrichment culture
DS: digester sample
HRT: hydraulic retention time
OLR: organic loading rate
F420+: F_420_ autofluorescence positive subcommunity
F420−: F_420_ autofluorescence negative subcommunity
F420 + S1: F_420_ autofluorescence positive subcommunity with lower SYBR Green I fluorescence
F420 + S2: F_420_ autofluorescence positive subcommunity with higher SYBR Green I fluorescence
