# Supplementary material for: Flow cytometric quantification, sorting and sequencing of methanogenic archaea based on F420 autofluorescence
Source: Microb Cell Fact. 2017 Oct 30;16:180. doi: 10.1186/s12934-017-0793-7 (PMC5663091; doi:10.1186/s12934-017-0793-7)
Supplement: Supplementary file 1 — Additional file 1. Additional information containing details about: S1 the sample origin, S2 the flow cytometer channel test, S3 the comparison of F420 fluorescent communities and a non-methanogenic control community, S4 the gating strategy used for the cell number determination, S5 the storage protocol testing, S6 the influence of sample storage on F420 fluorescence, S7 the nucleic acid staining, S8 the community analysis, S9 the sequencing protocols and details and S10 the digester screening. [file 12934_2017_793_MOESM1_ESM.docx]

# Additional information for

Flow cytometric quantification, sorting and sequencing of methanogenic archaea based on F_420_ autofluorescence

Johannes Lambrecht, Nicolas Cichocki, Thomas Hübschmann, Christin Koch, Hauke Harms and Susann Müller

Department of Environmental Microbiology, Helmholtz Centre for Environmental Research - UFZ, Permoserstr. 15, 04318 Leipzig, Germany

## Content:

S1**:** Sample origin 2

S2: Flow cytometer channel test 3

S3: F_420_ fluorescent communities vs. non-methanogenic control 3

S4: Gating strategy for cell number determination 4

S5: Storage protocol screening 5

S6: Influence of sample storage on F_420_ fluorescence 6

S7: Nucleic acid staining 10

S8: Community analysis 11

S9: Sequencing protocols and details 12

S10: Digester screening 15

## S1: Sample origin

### Methanogenic enrichment culture (MEC) medium

The MEC was cultivated on a modified complex DSMZ (Deutsche Sammlung von Mikroorganismen und Zellkulturen) medium 120a as described by Popp (2016). Ingredients were mixed under anoxic conditions (98% N_2_ and 2% H_2_) and 23.8 mL of the medium was added into 50-mL serum bottles. The composition of the medium was as follows: 2 g yeast extract, 1 g tryptone, 1 g coumarin, 0.348 g K_2_HPO_4_, 0.227 g KH_2_PO_4_, 0.5 g NH_4_Cl, 0.406 g MgCl_2_ x 6 H_2_O, 0.25 g CaCl_2_ x 2 H_2_O, 2.25 g NaCl, 1.42 mg FeCl_2_ x 4 H_2_O and 1 mg resazurin where mixed with 1 mL of trace element solution 10, consisting of 1.5 g L^-1^ FeCl_2_ x 4 H_2_O, 6 mg L^-1^ H_3_BO_3_, 190 mg L^-1^ CoCl_2_ x 6 H_2_O, 100 mg L^-1^ MnCl_2_ x 4 H_2_O, 70 mg L^-1^ ZnCl_2_, 36 mg L^-1^ Na_2_MoO_4_ x 2 H_2_O, 24 mg L^‑1^ NiCl_2_ x 6 H_2_O and 2 mg L^-1^ CuCl_2_ x 2 H_2_O. All components were dissolved in 960 mL aqua dest. and the pH set to ca. 4.0 with HCl. After sealing with butyl rubber stoppers and autoclaving, 0.1 mL sterile vitamin solution (DSM141), 0.5 mL sterile cysteine solution (30 mg L^-1^) and 0.5 mL sterile NaHCO_3_ solution (84 g L^-1^) were added. The pH value was adjusted to 7.5 - 7.9 by adding a sterile NaCO_3_ solution (50 g L^-1^). With 2 g yeast extract, 1 g tryptone, 1 g coumarin, the enrichment culture medium contained a total of 4 g L^-1^ complex substrate usable as a carbon and energy source.

### Digester sample (DS) substrate and digestate composition

Table S1: Feed and digestate composition of the main digester (DS). Acid spectrum and alcohol content was measured with headspace-GC (GC System 7890 A, Agilent Technologies, Santa Clara, California, USA using 2‑Methylbutyric acid as an internal standard). Dry mass and organic dry mass were gravimetrically obtained after drying at 105 °C and at 550 °C respectively.

|  | constituents | | | | | | Dry mass | Organic dry mass |
| --- | --- | --- | --- | --- | --- | --- | --- | --- |
|  | [mg L^-1^] | | | | | | [%] | [%_dm_] |
| **Feed** |  |  |  |  | Total acids | Total alcohols |  |  |
|  |  |  |  |  | 20874.1 | 332.6 | 8 | 83.23 |
|  |  |  |  |  |  |  |  |  |
| **Digestate** | Acetic acid | Propionic acid | i-Butyric acid | n-Butyric acid | i-Valeric acid | n-Caproic acid |  |  |
|  | 55 | 11.7 | 1.3 | 1.8 | 1.4 | 0.3 | 2.08 | 49 |

The digester feed contained a total of 66.58 g L^-1^ organic dry mass potentially usable as a carbon and energy source. It consisted of 8% total dry mass with 83.23% of organic constituents. The digester showed a volumetric methane productivity of 0.91 L_CH4_ L^‑1^ d^-1^ at an OLR of 2.0 g L^‑1^d^-1^. The digestate itself contained a total of 10.19 g L^-1^ organic dry mass just before the daily substrate input.

## S2: Flow cytometer channel test


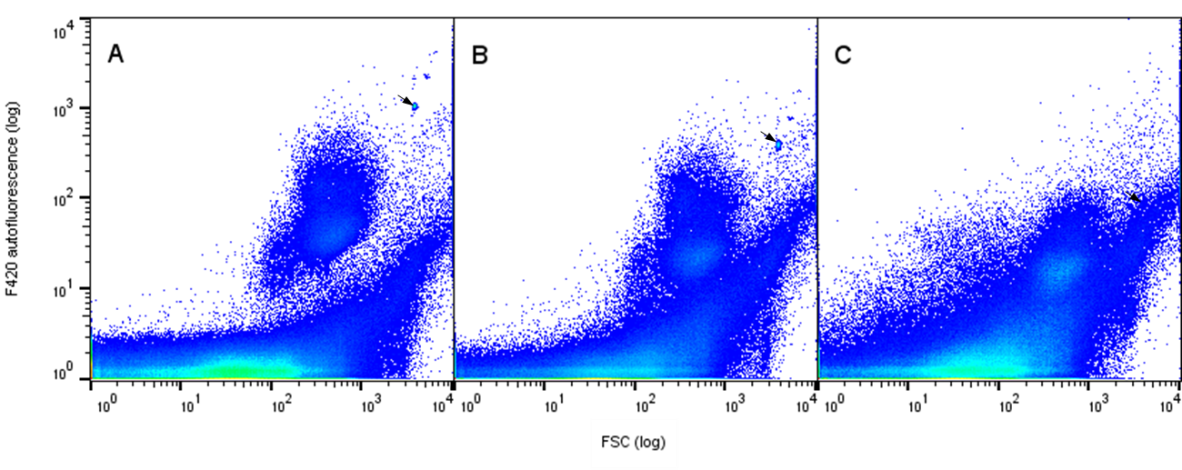


**Figure S2:** Comparison of flow cytometer channels for F_420_ fluorescence analysis showing in FSC vs. F_420_ autofluorescence of a fresh digester sample. The following excitation lines and fluorescence filter sets were used: **A:** 405 nm laser excitation and 460/50 nm fluorescence filter **B:** 405 nm laser excitation and 520/35 nm fluorescence filter and **C:** 355 nm laser excitation and 460/50 nm fluorescence filter. The arrow marks the added control beads. The best separation from non-autofluorescent cells was achieved for setting A.

**S3: F_420_ Fluorescent communities vs. non-methanogenic control**

**
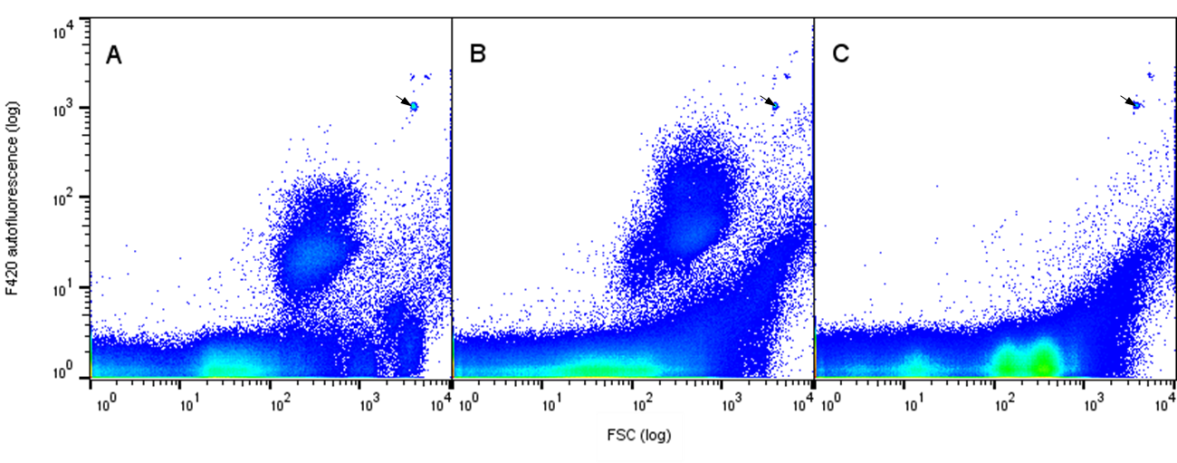
**

**Figure S3:** Presence or absence of methanogenic archaea in **A**: methanogenic enrichment culture, **B**: digester sample and **C**: microbial community of an anaerobic digester producing medium-chain fatty acids shown in FSC vs. F_420_ autofluorescent plots. The absence of subcommunity F420+ in C correlated with gas composition of the medium-chain fatty acids producing reactor (0% CH_4_, 68.7% CO_2_, 30.3% H_2_ and 1%N_2_). The arrow marks the added control beads.

**S4: Gating strategy for cell number determination**


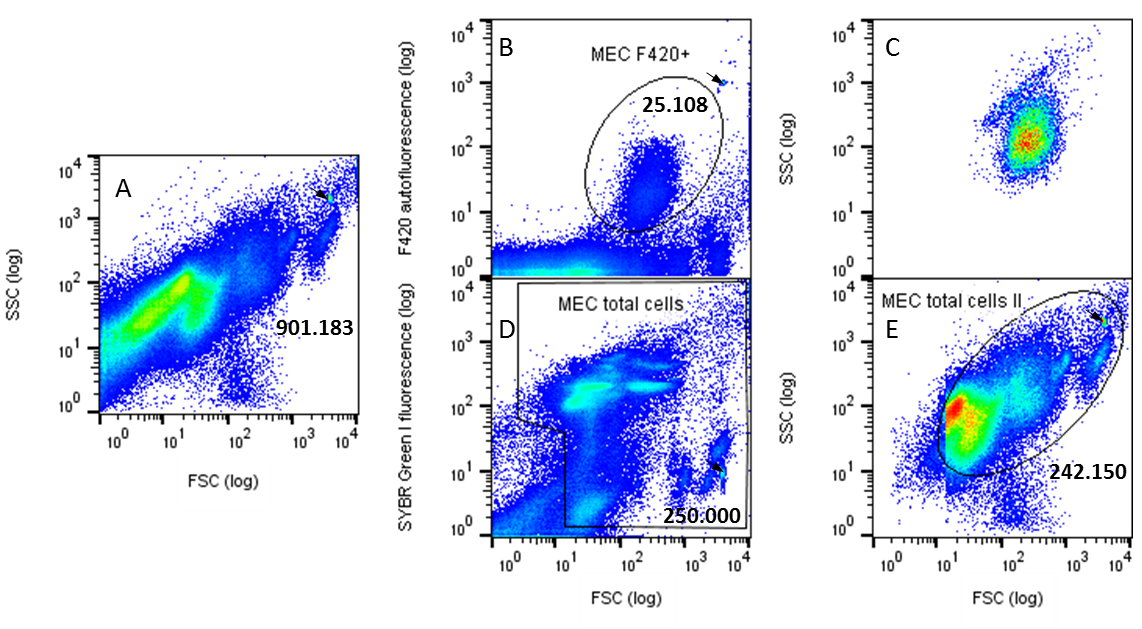


**Figure S4:** Visualization of the gating strategy applied for cell count determination. The numbers in the 2D-plots and gates represents the respective cell counts. **A**: All recorded events in a FSC vs. SSC 2D-plot. **B**: All recorded events in a FSC vs. F_420_ autofluorescence 2D-plot. The gate MEC F420+ determines the abundance of the subcommunity F420+. **C**: The subcommunity in MEC F420+ displayed in a FSC vs. SSC 2D-plot. **D**: All recorded events in a FSC vs. SYBR Green I fluorescence 2D-plot. The gate MEC total cells determines the abundance of all stained cells. **E**: The subcommunity MEC total cells in displayed in a FSC vs. SSC 2D-plot. The gate MEC total cells II can be created to determine the total cell count if no SYBR Green I staining is available. The arrow marks the added control beads which were excluded from the cell counting.

## S5: Storage protocol testing

**Figure 1 S5:** Comparison of different storage and fixation procedures on a digester sample based on ● relative abundances reated to the total cell number and ● mean F_420_ autofluorescence intensity of cells in gate F420+ (gating strategy see Figure S4). A **control** (fresh cells) is compared to its aliquots after three days of **1)** suspension in PBS at 6°C, **2)** fixation by 2% formaldehyde for 30 min at room temperature and suspension in PBS at 6°C after formaldehyde removal, **3)** fixation in the drying centrifuge IR MICRO-CENVAC NB-503CIR from (N-Biotec, Bucheon-si, South Korea) for 40 min at 35°C, 550 g, infrared radiation and storage of the pellets at 6 °C, **4)** suspension in a 15% Glycerol - PBS solution at 6 °C, **5)** suspension in 15% glycerol - PBS solution at – 20 °C. Each procedure was tested in three technical replicates and the error bars indicate the standard deviation of the replicates

**
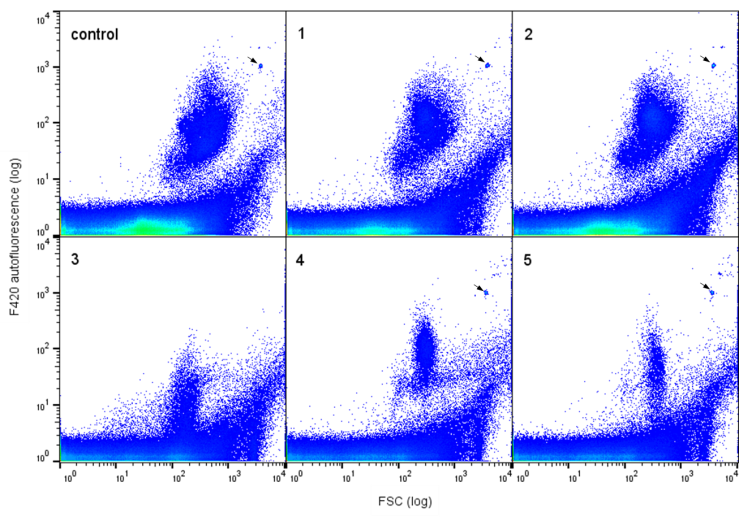
**

**Figure 2 S5:** Comparison of different storage and fixation procedures for a digester sample after 3 days shown in by FSC vs. F_420_ plots (exc. 405 nm, em. 460/50): **Control)** fresh cells, **1)** sample suspended in PBS at 6°C, **2)** fixation by 2% formaldehyde for 30 min at room temperature and suspended in PBS at 6 °C after formaldehyde removal, **3)** fixation in drying centrifuge IR MICRO-CENVAC NB-503CIR (N-Biotec, Bucheon-si, South Korea) for 40 min at 35°C, 550 g, infrared radiation and storage of the pellets at 6 °C, **4)** in 15% Glycerol / PBS solution at 6 °C and **5)** sample suspended in 15% glycerol / PBS solution at – 20 °C. 1,000,000 events where recorded for every measurement. The arrow marks the added control beads.

## S6: Influence of sample storage on F_420_ fluorescence


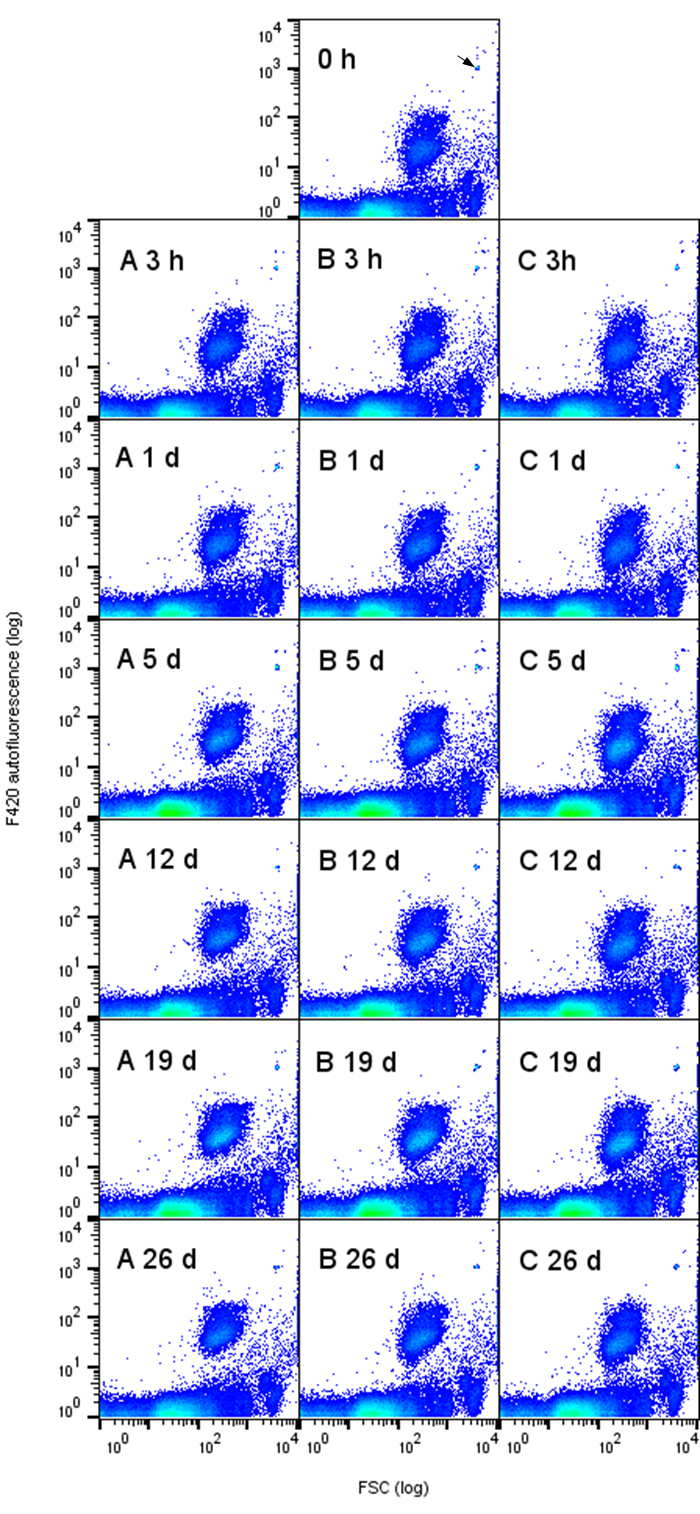


**Figure 1 S6:** Sample stability of F420+ in the MEC suspended in PBS over 26 days at **A:** Room temperature **B:** 6°C and **C:** 0°C. Each sample was analyzed with 500,000 events and is shown in a FSC vs. F_420_ 2D-plot. The arrow marks the control beads, added to every sample.

**Table 1 S6:** Sample stability of the MEC suspended in PBS over 26 days. Absolute abundance per mL and F_420_ autofluorescence intensity of F420+ and absolute abundance of F420- are given with their respective standard deviations. Samples were gated according to S4. Abundances and F_420_ autofluorescence intensities are visualized over time in the diagrams in Figure 2.

|  | **F420+** | | |
| --- | --- | --- | --- |
|  | 22 °C | 6 °C | 0 °C |
|  | cell number [mL^-1^]  (±Standard dev.) | cell number [mL^-1^]  (±Standard dev.) | cell number [mL^-1^]  (±Standard dev.) |
| fresh cells | 3.70 x 10^8^ (± 6.64 x 10^6^) |  |  |
| 3h | 3.82 x 10^8^ (± 1.05 x 10^7^) | 3.86 x 10^8^ (± 7.52 x 10^6^) | 3.69 x 10^8^ (± 3.12 x 10^6^) |
| 1d | 3.73 x 10^8^ (± 3.79 x 10^6^) | 3.86 x 10^8^ (± 1.62 x 10^7^) | 3.69 x 10^8^ (± 1.48 x 10^7^) |
| 5d | 3.89 x 10^8^ (± 1.05 x 10^7^) | 3.96 x 10^8^ (± 5.70 x 10^6^) | 4.01 x 10^8^ (± 1.97 x 10^7^) |
| 12d | 3.82 x 10^8^ (± 1.08 x 10^7^) | 3.94 x 10^8^ (± 2.22 x 10^7^) | 3.92 x 10^8^ (± 2.70 x 10^7^) |
| 19d | 3.89 x 10^8^ (± 1.25 x 10^7^) | 3.90 x 10^8^ (± 9.15 x 10^6^) | 4.01 x 10^8^ (± 2.61 x 10^7^) |
| 26d | 3.29 x 10^8^ (± 2.80 x 10^7^) | 3.55 x 10^8^ (± 6.33 x 10^6^) | 3.75 x 10^8^ (± 5.90 x 10^7^) |
|  |  | | |
|  | 22 °C | 6 °C | 0 °C |
|  | Autofluorescence intensity  (±Standard dev.) | Autofluorescence intensity  (±Standard dev.) | Autofluorescence intensity  (±Standard dev.) |
| fresh cells | 33.03 (± 0.12) |  |  |
| 3 h | 36.37 (± 0.35) | 32.27 (± 0.06) | 33.03 (± 0.06) |
| 1 d | 41.57 (± 0.21) | 39.73 (± 0.12) | 36.37 (± 0.06) |
| 5 d | 51.87 (± 0.15) | 44.37 (± 0.25) | 39.20 (± 0.35) |
| 12 d | 55.57 (± 0.23) | 47.40 (± 0.20) | 41.33 (± 0.25) |
| 19 d | 62.47 (± 0.87) | 54.87 (± 0.29) | 47.97 (± 0.59) |
| 26 d | 66.53 (± 1.14) | 56.90 (± 0.46) | 50.67 (± 0.65) |
|  |  | | |
|  | **F420-** | | |
|  | 22 °C | 6 °C | 0 °C |
|  | cell number [mL^-1^]  (±Standard dev.) | cell number [mL^-1^]  (±Standard dev.) | cell number [mL^-1^]  (±Standard dev.) |
| fresh cells | 3.47 x 10^9^ (± 6.36 x 10^7^) |  |  |
| 3 h | 3.49 x 10^9^ (± 4.54 x 10^7^) | 3.54 x 10^9^ (± 4.94 x 10^7^) | 3.38 x 10^9^ (± 8.42 x 10^7^) |
| 1 d | 3.42 x 10^9^ (± 5.66 x 10^7^) | 3.49 x 10^9^ (± 1.12 x 10^8^) | 3.37 x 10^9^ (± 1.11 x 10^8^) |
| 5 d | 3.80 x 10^9^ (± 6.90 x 10^7^) | 3.66 x 10^9^ (± 2.19 x 10^8^) | 3.62 x 10^9^ (± 1.70 x 10^8^) |
| 12 d | 3.75 x 10^9^ (± 1.68 x 10^8^) | 3.80 x 10^9^ (± 1.99 x 10^8^) | 3.83 x 10^9^ (± 2.64 x 10^8^) |
| 19 d | 3.42 x 10^9^ (± 5.04 x 10^7^) | 3.45 x 10^9^ (± 1.21 x 10^8^) | 3.30 x 10^9^ (± 3.26 x 10^8^) |
| 26 d | 2.94 x 10^9^ (± 2.43 x 10^8^) | 3.11 x 10^9^ (± 8.53 x 10^7^) | 3.22 x 10^9^ (± 2.72 x 10^8^) |


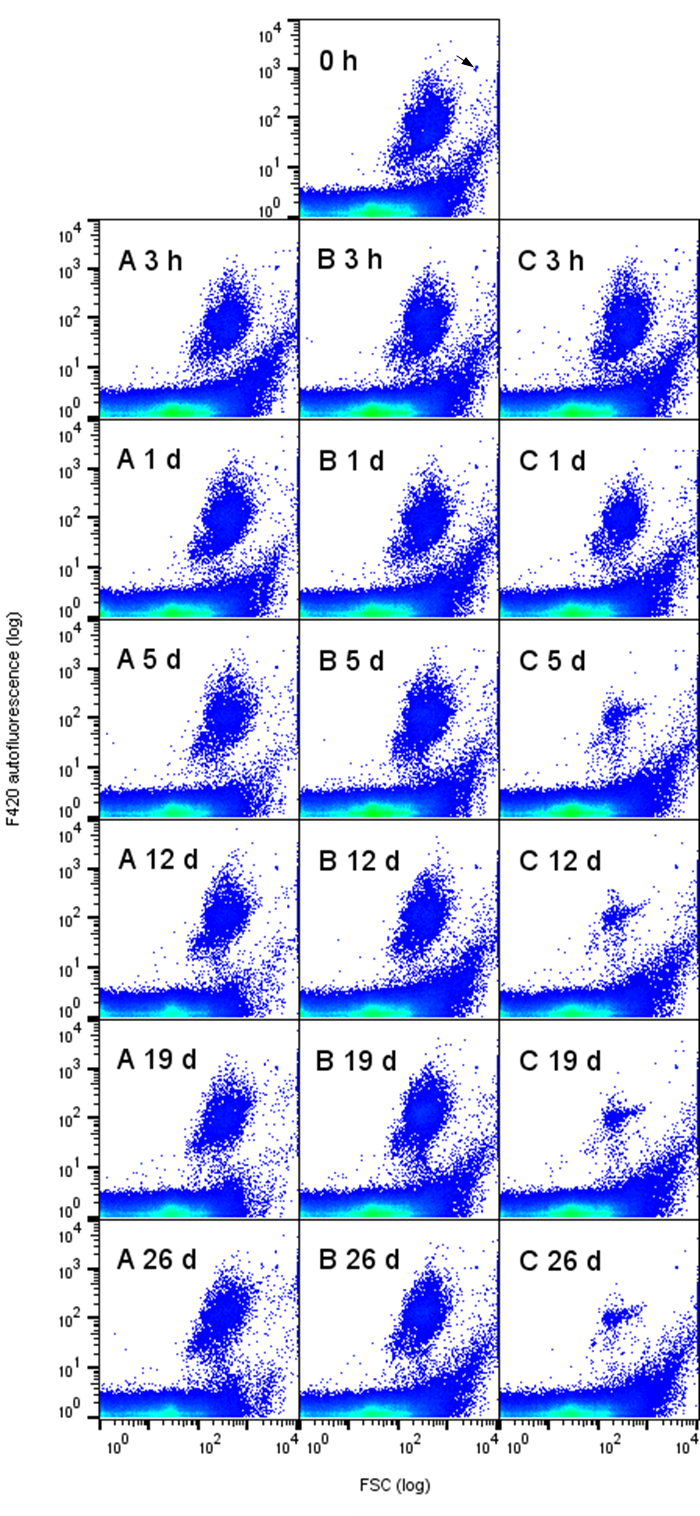


**Figure 2 S6:** Stability of F420+ in the digester sample suspended in PBS over 26 days at **A** 22 °C, **B** 6 °C and **C** 0 °C. Each sample was analyzed with 1,000,000 events and is shown in a FSC vs. F_420_ 2D-plot. The arrow marks the control beads, added to every measurement.

**Table 2 S6:** Sample stability of a digester sample suspended in PBS over 26 days. Absolute abundance per mL and F_420_ autofluorescence intensity of F420+ and absolute abundance of F420- are given with their respective standard deviations. Samples were gated according to S4. Abundances and F_420_ autofluorescence intensities are visualized over time in the diagrams in Figure 2.

|  | **F420+** | | |
| --- | --- | --- | --- |
|  | 22 °C | 6 °C | 0 °C |
|  | cell number [mL^-1^]  (±Standard dev.) | cell number [mL^-1^]  (±Standard dev.) | cell number [mL^-1^]  (±Standard dev.) |
| fresh cells | 1.81 x 10^9^ (± 2.27 x 10^8^) |  |  |
| 3h | 1.67 x 10^9^ (± 1.35 x 10^8^) | 1.49 x 10^9^ (± 7.85 x 10^7^) | 1.34 x 10^9^ (± 2.36 x 10^7^) |
| 1d | 1.68 x 10^9^ (± 1.14 x 10^8^) | 1.73 x 10^9^ (± 1.56 x 10^8^) | 9.40 x 10^8^ (± 7.15 x 10^7^) |
| 5d | 1.56 x 10^9^ (± 2.24 x 10^8^) | 1.53 x 10^9^ (± 1.12 x 10^8^) | 1.75 x 10^8^ (± 3.95 x 10^6^) |
| 12d | 1.51 x 10^9^ (± 2.26 x 10^8^) | 1.59 x 10^9^ (± 1.34 x 10^8^) | 1.79 x 10^8^ (± 5.68 x 10^6^) |
| 19d | 1.39 x 10^9^ (± 2.71 x 10^7^) | 1.47 x 10^9^ (± 1.35 x 10^8^) | 1.52 x 10^8^ (± 1.21 x 10^7^) |
| 26d | 1.22 x 10^9^ (± 3.06 x 10^8^) | 1.51 x 10^9^ (± 1.74 x 10^8^) | 1.24 x 10^8^ (± 7.31 x 10^6^) |
|  |  | | |
|  | 22 °C | 6 °C | 0 °C |
|  | Autofluorescence intensity  (±Standard dev.) | Autofluorescence intensity  (±Standard dev.) | Autofluorescence intensity  (±Standard dev.) |
| fresh cells | 93.00 (± 2.52) |  |  |
| 3 h | 103.67 (± 1.53) | 108.67 (± 1.15) | 101.00 (± 0.00) |
| 1 d | 121.67 (± 7.37) | 116.67 (± 3.79) | 110.00 (± 0.00) |
| 5 d | 137.67 (± 5.03) | 135.67 (± 2.08) | 95.23 (± 0.91) |
| 12 d | 141.67 (± 11.06) | 147.67 (± 4.62) | 94.77 (± 1.27) |
| 19 d | 142.67 (± 10.60) | 155.00 (± 4.58) | 92.50 (± 0.46) |
| 26 d | 147.33 (± 6.11) | 152.33 (± 5.13) | 88.23 (± 1.33) |
|  |  | | |
|  | **F420-** | | |
|  | 22 °C | 6 °C | 0 °C |
|  | cell number [mL^-1^]  (±Standard dev.) | cell number [mL^-1^]  (±Standard dev.) | cell number [mL^-1^]  (±Standard dev.) |
| fresh cells | 3.96 x 10^10^ (± 6.51 x 10^9^) |  |  |
| 3 h | 3.81 x 10^10^ (± 5.97 x 10^9^) | 2.94 x 10^10^ (± 1.42 x 10^9^) | 2.87 x 10^10^ (± 1.73 x 10^8^) |
| 1 d | 3.64 x 10^10^ (± 3.74 x 10^9^) | 3.71 x 10^10^ (± 4.61 x 10^9^) | 2.91 x 10^10^ (± 2.55 x 10^9^) |
| 5 d | 3.83 x 10^10^ (± 7.32 x 10^9^) | 3.97 x 10^10^ (± 5.01 x 10^9^) | 2.70 x 10^10^ (± 5.86 x 10^8^) |
| 12 d | 3.09 x 10^10^ (± 3.03 x 10^9^) | 3.80 x 10^10^ (± 7.07 x 10^9^) | 2.81 x 10^10^ (± 1.39 x 10^9^) |
| 19 d | 2.90 x 10^10^ (± 3.55 x 10^9^) | 3.69 x 10^10^ (± 6.74 x 10^9^) | 2.86 x 10^10^ (± 1.21 x 10^9^) |
| 26 d | 2.22 x 10^10^ (± 6.26 x 10^9^) | 3.55 x 10^10^ (± 5.01 x 10^9^) | 2.72 x 10^10^ (± 1.36 x 10^9^) |

## S7: Nucleic acid staining

**
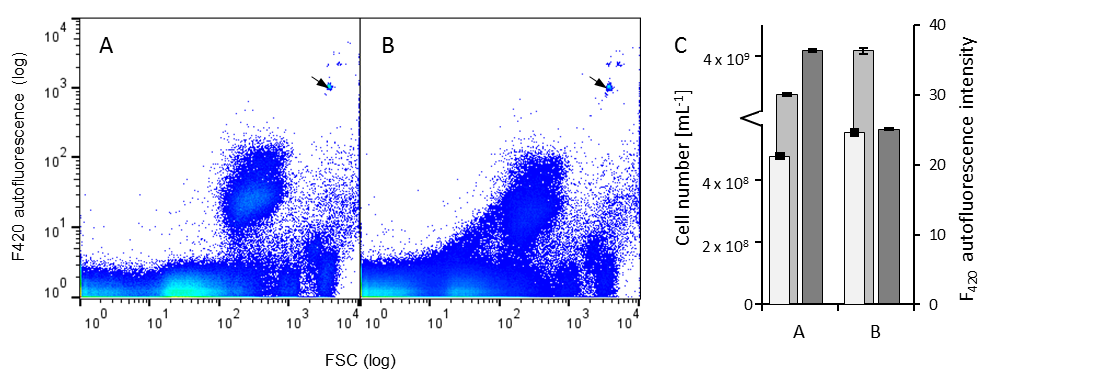
**

**Figure S7:** Influence of SYBR Green I staining on F_420_ fluorescence of the MEC. **A**: Unstained methanogenic enrichment culture after 3 h as a control. **B:** The same sample after 3 h of staining. **C:** Cell numbers in F420+ (●), F420- (●) and autofluorescence intensity (●) of F420+ are indicated with the respective standard deviations. Samples were gated according to S4. Values are given in Table 1 S7. The arrow marks the added control beads.

**Table 1 S7:** Influence of nucleic acid staining on F_420_ fluorescence of the MEC. Cell number and fluorescence intensity in F420+ and abundances in F420- are indicated with the respective standard deviations.

|  | **F420+** | |  |
| --- | --- | --- | --- |
|  | A unstained | B stained (SYBR Green I) | |
| Cell number [mL^-1^]  (±Standard dev.) | 4.78 x 10^8^ (± 1.31 x 10^7^) | 5.56 x 10^8^ (± 1.64 x 10^7^) | |
| Autofluorescence intensity  (±Standard dev.) | 36.37 (± 0.35) | 25.13 (± 0.15) | |
|  | **F420-** | |  |
| Cell number [mL^-1^]  (±Standard dev.) | 3.93 x 10^9^ (± 4.31 x 10^7^) | 4.09 x 10^9^ (± 9.82 x 10^7^) | |

**Table 2 S7:** Influence of nucleic acid staining on F_420_ fluorescence of a digester sample. Cell number and fluorescence intensity in F420+ and abundances in F420- are indicated with the respective standard deviations.

|  | **F420+** | |  |
| --- | --- | --- | --- |
|  | A unstained | B stained (SYBR Green I) | |
| Cell number [mL^-1^]  (±Standard dev.) | 1.70 x 10^9^ (± 1.36 x 10^8^) | 1.93 x 10^9^ (± 2.39 x 10^8^) | |
| Autofluorescence intensity  (±Standard dev.) | 103.67 (± 1. 53) | 75.53 (± 3.52) | |
|  | **F420-** | |  |
| Cell number [mL^-1^]  (±Standard dev.) | 3.02 x 10^10^ (± 4.78 x 10^9^) | 3.34 x 10^10^ (± 5.53 x 10^9^) | |

## S8: Community structure

**
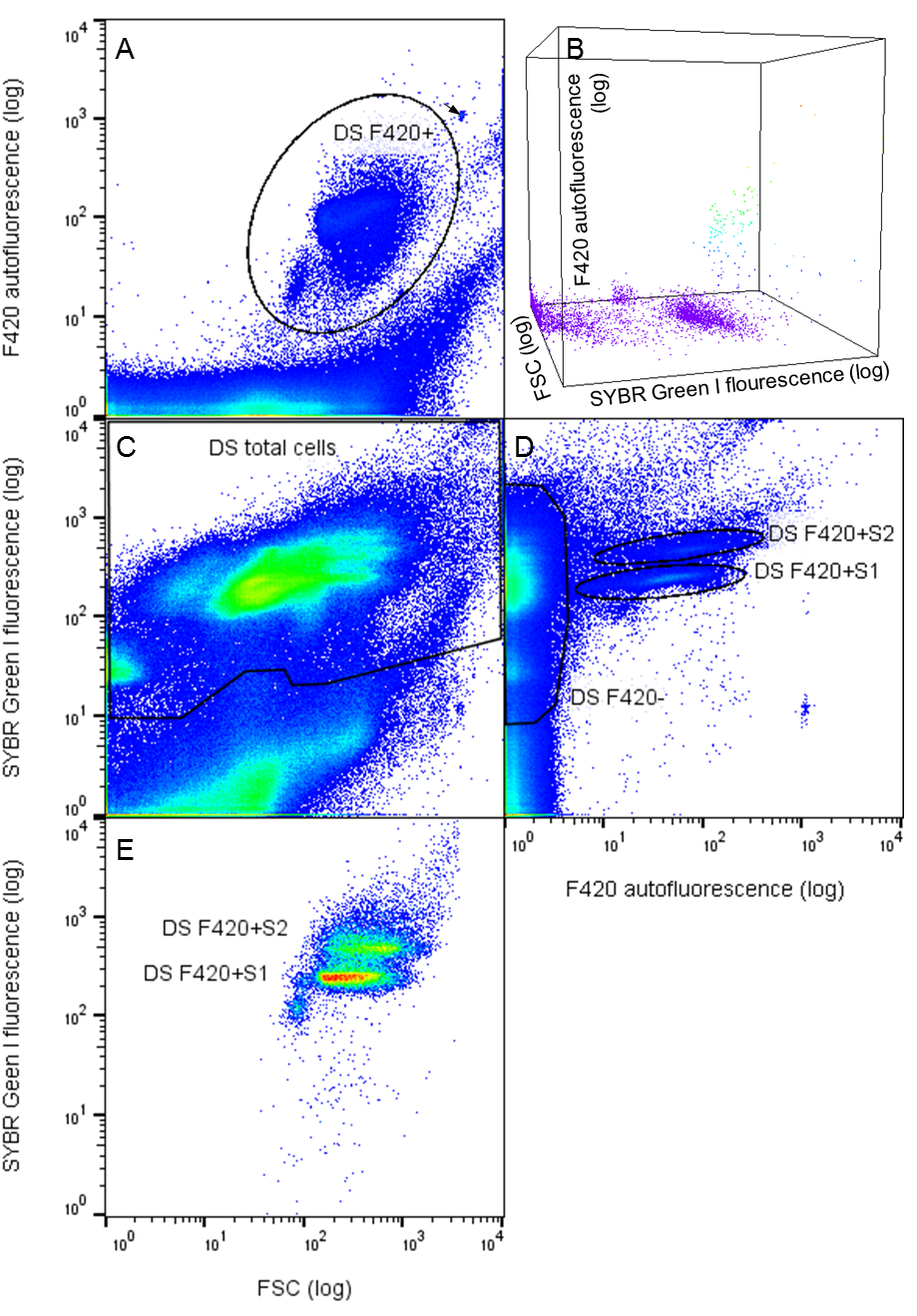
**

**Figure S8:** Flow cytometric analysis of a digester sample (DS) with sort gates in black (**A** unstained, **B**, **C**, **D**, **E** stained). **A:** FSC vs. F_420_ plot, with the high autofluorescent subcommunity in gate: DS F420+. **B:** 3D visualization of the channels of interest, FSC vs. F_420_ vs. SYBR Green I. **C:** FSC vs. SYBR Green I plot with the whole microbial community in the gate: DS total cells. **D:** F_420_ vs SYBR Green I plot with the non-autofluorescent community in gate: DS F420- and the F_420_ fluorescent subcommunities in the gates: DS F420+S1 and DS F420+S2. **E:** Position of these two subcommunities in a FSC vs. SYBR Green I plot. The arrow marks the control beads.

**Table 1 S8:** Mean intensity values of the subcommunities of the DS. The gates are defined in Figure S8.

| Gate | Mean channel intensity | | |
| --- | --- | --- | --- |
|  | FSC | F_420_ autofluorescence | SYBR Green I fluorescence |
| DS F420+ | 418 | 103 |  |
| DS total cells | 152 | 5.59 | 261 |
| DS F420- | 108 | 1.29 | 224 |
| DS F420+S1 | 422 | 47.3 | 242 |
| DS F420+S2 | 699 | 82.9 | 496 |

**Table 2 S8:** Mean intensity values of the subcommunities of the MEC. The gates are defined in Figure 4.

| Gate | Mean channel intensity | | |
| --- | --- | --- | --- |
|  | FSC | F_420_ autofluorescence | SYBR Green I fluorescence |
| MEC F420+ | 398 | 35.8 |  |
| MEC F420low | 3122 | 4.44 |  |
| MEC total cells | 273 | 3.39 | 231 |
| MEC F420- & MEC F420low | 234 | 1.48 | 211 |
| MEC F420+S1 | 409 | 26.5 | 226 |
| MEC F420+S2 | 517 | 38.5 | 442 |

**S9: Sequencing protocols and details**


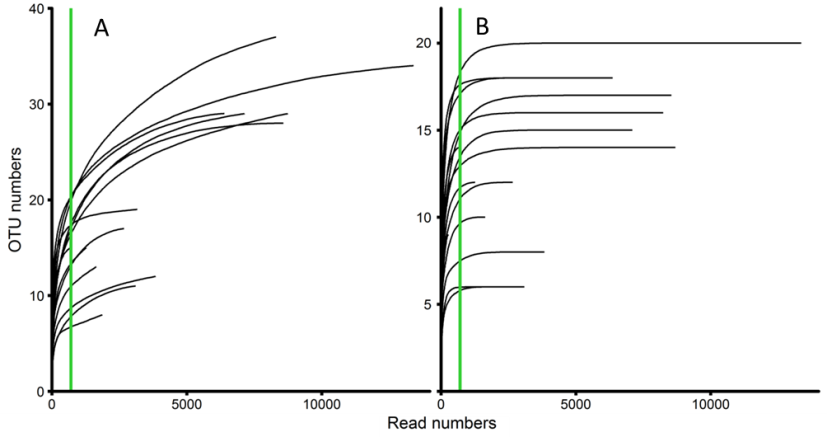


**Figure S9:** Rarefaction analysis of *mcrA* amplicon sequencing reads of all 15 samples with **A:** Raw data and **B:** Processed data with an OTU threshold of 0.1%. The green line indicates the chosen normalization threshold at 701 reads.

**Table S9:** Processed *mcrA* sequence specific read numbers of the sorted subcommunities of the methanogenic enrichment culture (MEC) and the two digester samples (DS1, DS2).

| sample | gate | | | | |
| --- | --- | --- | --- | --- | --- |
|  | total cells | F420- | F420+ | F420+S1 | F420+S2 |
| MEC | 1641 | 1868 | 1271 | 3103 | 3840 |
| DS1 | 2669 | 701 | 13402 | 8309 | 7136 |
| DS2 | 3166 | 277 | 8579 | 8742 | 6395 |

### DNA Extraction and quality testing

The DNA extraction procedure was performed according to (Koch et al., 2013). 70 µL 10% (wt/vol) Chelex 100 solution (Biorad, [Hercules California](https://en.wikipedia.org/wiki/Hercules,_California), [U.S.A.](https://en.wikipedia.org/wiki/United_States)) was used to extract DNA from 500,000 cells before storage at - 20 °C. The quality of the extracted DNA was tested by visualizing the product of a preceding 35 cycle PCR via gel electrophoresis (1.5% agarose). The PCR utilized the *mcrA* primer pair MLF 5’‑GGTGGTGTMGGATTCACACARTAYG CWACAGC-3’ and MLR 5’-TTCATTGCRTAGTTWGGRTAGTT-3’ described by Luton et al. (2002) and was performed using a S1000 Thermal cycler (Biorad) like all the following PCRs.

### Library preparation for Illumina® MiSeq sequencing technique

We used metaprofiling of the *mcrA* gene coding for the α-subunit of the methyl coenzyme M reductase to investigate the methanogenic population in the subcommunities. The isolated DNA was used to create the library for the MiSeq Illumina® sequencing run by utilizing the primer pair mentioned above (MLF, MLR). To process a large number of samples in one run, dual-barcoding was performed according to the sequencing company’s protocol (Fasteris SA, Plan-les-Ouates, Switzerland). The barcoded primers (5’-barcode-mother primer-3’) used CT, AT, GT and TGAT as forward and GC, TG, CA and AA as reverse barcodes. The barcodes were chosen as short as possible due to the already considerable length of the mother primers. A double step PCR with 20 cycles of the mother primers and 10 cycles of the barcoded primers was performed. The PCR products were purified with the Agencourt® AMPure® XP-Kit (Beckman Coulter, Brea, California, USA) between and after the two steps. The amplicons were quantified with Qubit 3.0 (Life technologies, Carlsbad, Califorania, USA) and finally pooled together to 30 µL in equimolar proportion to be sequenced.

The PCRs were performed in 10 μL batches, containing 10 pmol of forward and reverse primers (Eurofins Scientific, Luxembourg City, Luxembourg), 2 nmol of dNTP mix (Promega, Fitchburg, Wisconsin, USA), 2 µL 5x Phusion® GC solution, 20 nmol of MgCl_2_, (both provided in the polymerase kit), 0.2 units of Phusion® High-Fidelity Polymerase (New England Biolabs, Ipswich, Massachusetts, USA) and 1 µL DNA solution. Nuclease free water (Qiagen, Velno, Netherlands) was added and adjusted the final reaction volume. The PCRs were performed under the following conditions: 3 min of initial denaturation at 95 °C, 30 sec denaturation at 95 °C, 60 sec annealing at 55 °C, 1 min extension at 72 °C and finally 10 min extension at 72 °C before storage at 4°C. For each reaction, a negative control without any DNA was amplified on 35 cycles and visualized via gel electrophoresis (1.5% agarose) to ensure absence of contamination. The library preparation step was done in triplicates for each sample. The triplicates were combined in equimolar proportion before the final pooling. The sequencing was performed on the MiSeq platform (Illumina, [San Diego, California](https://en.wikipedia.org/wiki/San_Diego,_California), [USA](https://en.wikipedia.org/wiki/United_States)) from the pooled sample by Fasteris. The sequencing run used the 2 x 300 bp, 600 cycles option with the v3 kit (Illumina), the meta fast library and the ATCACG index (Illumina).

### Sequencing data evaluation

The quality filtering was set to q = 27. The Mothur version 1.36 (Schloss et al., 2009) was used for Illumina library demultiplexing, merging the forward and reverse sequences, removing singletons, normalizing the dataset and OTU classification. UCHIME (Edgar et al., 2011) was used for removing chimeras. Rarefaction curves were plotted using ggplot2 package in R (Wickham, 2009).

The analysis based on a data set of 71.099 forward-reverse overlapped sequences. *Pseudomonas putida* KT2440 was introduced to the sequencing run as a mock strain and positive control that was detected and correctly assigned in 4.705 of 4.723 reads (99.7%). 14 of the 15 original samples were subsampled to 701 reads during normalization (one sample yielded only 277 reads). The OTU assignment was performed using the mcrA data base created by Yang et al. (2014) (http://fungene.cme.msu.edu/hmm_detail.spr?hmm_id=16) with the average neighbor clustering algorithm at 97% sequence similarity cut off. After applying the minimum threshold of 0.1% sequence abundance, the gamma diversity of the whole dataset comprised of 53 OTUs while the normalized dataset contained 28 OTUs. As the presented method is not focusing on the ecology of the rare biosphere but the quantification of biotechnological relevant methanogenic archaea, the data analysis is focused only on the more abundant organisms with an OTU threshold of over 0.1%.

## S10: Digester screening

**
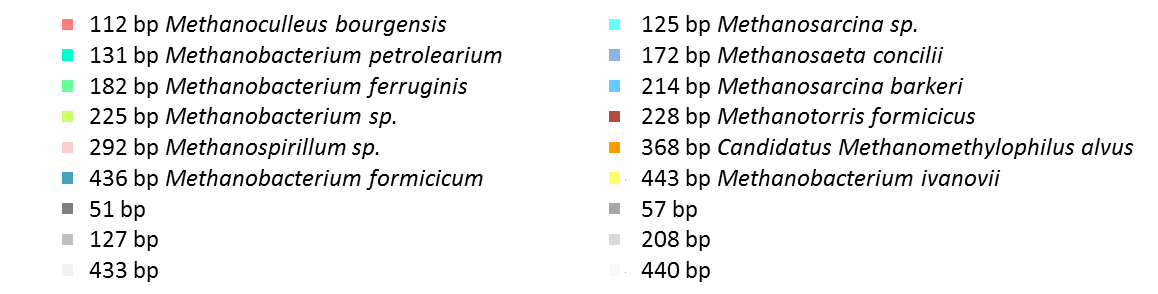
**

**Figure S 10:** Community fingerprint of the screened digesters A to H including T-RFs with an abundancy of over 1% (for process parameters, F420+ cell numbers and fluorescence intensities see table 1). The mcrA targeted T-RFLP analysis of methanogenic archaea in the fresh samples is shown for each digester.
